# Comparing methods for comparing networks

**DOI:** 10.1038/s41598-019-53708-y

**Published:** 2019-11-26

**Authors:** Mattia Tantardini, Francesca Ieva, Lucia Tajoli, Carlo Piccardi

**Affiliations:** 1 Moxoff SpA, via Schiaffino 11/A, 20158 Milano, Italy; 2 MOX - Modelling and Scientific Computing Lab, Department of Mathematics, Politecnico di Milano, Via Bonardi 9, 20133 Milano, Italy; 3 CADS - Center for Analysis, Decisions and Society, Human Technopole, 20157 Milano, Italy; 4 Department of Management, Economics and Industrial Engineering, Politecnico di Milano, Via Lambruschini 4/b, 20156 Milano, Italy; 5 Department of Electronics, Information and Bioengineering, Politecnico di Milano, Piazza Leonardo da Vinci 32, 20133 Milano, Italy

**Keywords:** Complex networks, Computational science

## Abstract

With the impressive growth of available data and the flexibility of network modelling, the problem of devising effective quantitative methods for the comparison of networks arises. Plenty of such methods have been designed to accomplish this task: most of them deal with undirected and unweighted networks only, but a few are capable of handling directed and/or weighted networks too, thus properly exploiting richer information. In this work, we contribute to the effort of comparing the different methods for comparing networks and providing a guide for the selection of an appropriate one. First, we review and classify a collection of network comparison methods, highlighting the criteria they are based on and their advantages and drawbacks. The set includes methods requiring known node-correspondence, such as DeltaCon and Cut Distance, as well as methods not requiring a priori known node-correspondence, such as alignment-based, graphlet-based, and spectral methods, and the recently proposed Portrait Divergence and NetLSD. We test the above methods on synthetic networks and we assess their usability and the meaningfulness of the results they provide. Finally, we apply the methods to two real-world datasets, the European Air Transportation Network and the FAO Trade Network, in order to discuss the results that can be drawn from this type of analysis.

## Introduction

The research on complex networks has exploded in the last two decades, thanks to the great power and flexibility of network models in describing real-world applications coming from very diverse scientific and technological areas, including social sciences, economics, biology, computer science, telecommunications, transportation, and many others^1–3^. A typical data-driven study begins with modelling or interpreting real-world data with a network, and then continues by exploiting a broad set of algorithmic tools, in order to highlight a number of network features from the local scale (nodes and edges) to the global scale (the overall network), passing through an intermediate (meso-)scale where the important structure and role of suitable subnetworks is possibly evidenced^4,5^.

With the dramatic growth of available data, and the corresponding growth of network models, researchers often face the problem of comparing networks, i.e., finding and quantifying similarities and differences between networks. In whatever application area, it is straightforward to find relevant examples where this problem is truly of interest: in international economics, one may want to compare the trade structure of different product categories; in transportation, the flight networks of different airlines; in biology, the interaction structure of different protein complexes; in social media, the propagation cascade of news; etc. Or, when temporally-stamped data are available, one may want to spot anomalous instants in a temporal series of graphs–e.g., an event that abruptly modifies the connection pattern among the users of a social media. Defining metrics for quantifying the similarity/difference between networks opens the door to clustering, i.e., grouping networks according to their similarity: for example, given a set of brain networks derived from NMR data, find whether and how ill-subjects’ networks are different from healthy-subjects’ ones.

To perform network comparison, a measure of the distance between graphs needs to be defined. This is a highly non trivial task, which requires a trade off among effectiveness of the results, interpretability, and computational efficiency, all features that, in addition, are often sensitive to the specific domain of application. Not surprisingly, the literature on this topic is extremely abundant, and plenty of different methods have been proposed. Yet much work has still to be done in terms of their comparative analysis, namely classifying which methods are the best and in which situations, and understanding the insights they can provide when used to compare a set of real-world networks. A few critical reviews of the literature on this subject have already been compiled (e.g., Soundarajan *et al*.^6^, Emmert-Streib *et al*.^7^, and Donnat and Holmes^8^). Our aim is to complement these works by systematically testing many of the available methods in a comparative fashion.

This paper is organised as follows. In the next section, we discuss the general problem of network comparison and we recall the definition and main features of many of the methods available in the literature, from naїve approaches to a few of the most recent ones. This gives an overview of the state-of-the-art tools and of the strategies for network comparison. Then we restrict our attention to a subset of such methods, and we analyse them with a suitably designed battery of tests on synthetic networks, in order to highlight pros and cons of each method. We subsequently illustrate two examples of the use of network comparison methods on real-world data, specifically the European Air Transportation Network and the FAO Trade Network. We conclude by summarizing the results of our work and by highlighting which methods reveal to be the most effective and in which situations. All the analyses are carried out using the codes made available by the authors of each method (see the Supplementary Information file, Sec. S2, for details).

## Measuring the Distance Between Networks

The *network comparison* problem derives from the *graph isomorphism* problem. Two (undirected, unweighted) graphs *G*_1_(*V*_1_, *E*_1_) and *G*_2_(*V*_2_, *E*_2_) are isomorphic if there exists a one-to-one correspondence Φ mapping the node set *V*_1_ onto *V*_2_ such that the edge (*u*, *v*) ∈ *E*_1_ if and only if the edge (Φ(*u*), Φ(*v*)) ∈ *E*_2_^9^. The complexity of the *graph isomorphism problem*, i.e., checking whether two finite graphs are isomorphic, is unknown in rigorous terms^10–12^: nonetheless, efficient algorithms exist for many classes of graphs^13^. In any case, isomorphism is an *exact graph matching*: if used as a distance for comparison, it gives a binary outcome: the graphs are either isomorphic, i.e., identical, or not. This information is poor, however, because networks are almost never identical in applications, and one is interested in assessing to what extent they are similar. To effectively compare networks, we need to move to *inexact graph matching*, i.e., define a real-valued distance which, as a minimal requirement, has the property of converging to zero as the networks approach isomorphism.

Searching for accurate and effective tools to compare networks has pushed the research in many different directions, leading to a wide variety of methods and algorithms. We present a short review of several of the most used approaches to network comparison, acknowledging that the literature is very abundant and we cannot cover it exhaustively neither want to repeat already established results. Following previous approaches^6^, we partition the comparison methods based on whether the induced distances are dependent from the correspondence of nodes. In the former case–*Known Node-Correspondence* (*KNC*)–the two networks have the same node set (or at least a common subset), and the pairwise correspondence between nodes is known. Thus, typically, only graphs of the same size and coming from the same application domain can be compared. In the latter case–*Unknown Node-Correspondence* (*UNC*)–ideally any pair of graphs (even with different sizes, densities, or coming from different application fields) can be compared: typically these methods summarize the global structure into one or more statistics, which are then elaborated to define a distance. This latter notion of distance thus reflects the difference in the global structure of the networks.

For example, imagine that one wants to compare the European air transportation networks of the airlines A and B. The node sets (i.e., the European airports) are the same, thus a KNC method can be applied to quantify to what extent the two sets of edges are similar, i.e., to what extent the two airlines offer the same set of flights. If the exercise is (pairwise) extended to all airlines, the overall results will allow one to cluster airlines supplying similar sets of connections. But the same dataset could alternatively be analysed, with a different aim, by a UNC method, to highlight pairs of airlines whose network is globally structurally similar. Then, for example, one might discover that airlines A and B have both a markedly star-like flight network, but the first is based in Amsterdam (the centre of the star) and the second in Berlin. Here, extending the analysis to all airlines might cluster sets of airlines with similar strategies or business models.

A screening of the literature reveals that the latter problem is far more studied–and that biology is the most popular application field–so that the number of available UNC methods is much larger than that of the KNC ones. Below we present several methods used for network comparison, briefly explaining the details of their approach. In doing that, we will mostly privilege methods of general use, that is, applicable to directed and weighted networks too–this significantly narrows the set of candidates. In the next section, we will numerically compare the performance of a subset of these methods.

### Known node-correspondence (KNC) methods

#### Difference of the adjacency matrices

The simplest and näive measures are obtained by directly computing the difference of the adjacency matrices of the two networks. Then any matrix norm can be used, e.g., Euclidean, Manhattan, Canberra, or Jaccard^14^. All of them are suitable to compare all kinds of graphs (directed or not, weighted or not), with the exception of the Jaccard distance which needs to be extended to the Weighted Jaccard distance^15^ (the definitions of the four norms are recalled in the Supplementary Information file, Sec. S1). Although this straightforward approach is rarely used in network comparison, we include it in the pool and consider it as a baseline approach.

#### DeltaCon

It is based on the comparison of the similarities between all node pairs in the two graphs^16,17^. The similarity matrix of a graph is defined by *S* = [*s*_*ij*_] = [*I* + *ε*^2^*D* − *εA*]^−1^, where *A* is the adjacency matrix, *D* = diag(*k*_*i*_) is the degree matrix, *k*_*i*_ is the degree of node *i*, and *ε* > 0 is a small constant. The rationale of the method is that just measuring the overlap of the two edge sets might not work in practice, because not all edges have the same importance. Instead, the difference between *r*-step paths, *r* = 2, 3, …, provides a much more sensitive measure. As a matter of fact, it can be shown^16^ that *s*_*ij*_ depends on all the *r*-paths connecting (*i*, *j*). The DeltaCon distance between the *N* × *N* similarity matrices *S*^1^ = [*s*_*ij*_^1^] and *S*^2^ = [*s*_*ij*_^2^] is finally defined using the Matusita distance:

1 $$d={(\mathop{\sum }\limits_{i,j=1}^{N}{(\sqrt{{s}_{ij}^{1}}-\sqrt{{s}_{ij}^{2}})}^{2})}^{1/2}.$$

Equation (1) assures that DeltaCon satisfies the usual axioms of distances. Moreover, it can be shown^16,17^ that it also satisfies a few desirable properties regarding the impact of specific changes. Such properties are: changes leading to disconnected graphs are more penalized; in weighted graphs, the bigger the weight of the removed edge is, the greater the impact on the distance; a change has more impact in low density graphs than in denser graphs with equal size; random changes produce smaller impacts than targeted ones.

The computational complexity of the DeltaCon algorithm is quadratic in the number of nodes. To improve execution speed, an approximated version was proposed, which restricts the computation of the similarity matrices to groups of randomly chosen nodes^16^: this version has linear complexity in the number of edges and groups. Finally, DeltaCon was extended^17^ to make it able to find which nodes or edges are most responsible for the differences between the two graphs.

#### Cut distance

 This method^12^ is based on the notion of cut weight, which is standard in graph theory and also used in community detection^4^. Given a (possibly directed, weighted) graph *G* = (*V*, *E*) with edge weights *w*_*ij*_, *i*, *j* ∈ *V*, and two disjoint node sets *S*, *T* ⊂ *V*, the cut weight is defined as ${e}_{G}(S,T)={\sum }_{i\in S,j\in T}\,{w}_{ij}$, i.e., the total weight of the edges crossing the cut from *S* to *T*. The cut distance between two graphs *G*_1_(*V*, *E*_1_) and *G*_2_(*V*, *E*_2_) with the same node set is then defined as

2 $$d({G}_{1},{G}_{2})=\mathop{{\rm{\max }}}\limits_{S\subset V}\frac{1}{|V|}|{e}_{{G}_{1}}(S,{S}^{C})-{e}_{{G}_{2}}(S,{S}^{C})|,$$

where *S*^*C*^ = *V*\*S*. Thus two networks are similar if they have similar cut weight for all possible network bipartitions. The maximization is performed through genetic algorithms, which makes the comparison of large networks (thousands of nodes and larger) unfeasible. On the other hand, this is one of the few methods able to compare directed, weighted graphs.

### Unknown node-correspondence (UNC) methods

#### Global statistics

Simple metrics can be obtained by comparing the value of network statistics, such as the clustering coefficient^18–21^, the diameter^19,21^, or the average distance^18^. Although being intuitive and typically computationally efficient, often this approach does not yield robust results. As a matter of fact, similar values of network statistics do not necessarily imply similar network structures (e.g., see the discussion in ref. ^22^) and indeed, the comparison often fails in catching important local features. On the other hand, these simple metrics offer a computationally low-cost alternative that can be useful for a first analysis.

#### Mesoscopic response functions (MRFs)

This method exploits the information carried by the mesoscopic properties of the networks, i.e., their modular structure^23^. Three functions–called MRFs–are defined, the Hamiltonian *H*(*λ*), the partition entropy *S*(*λ*), and the number of communities *η*(*λ*), which describe the properties of a given network at different mesoscopic scales: the parameter *λ* tunes the fragmentation of the network into communities. The network distance is defined for a given set of graphs: for each network pair, the distances between corresponding MRFs are defined by standard function metrics, then the first principal component obtained from PCA is taken as distance. This entails non-comparability among different datasets, since the distance between two networks depends on the dataset they are part of. On the other hand, it is the only available method based on mesoscale properties, and allows one to consider both weighted and unweighted undirected networks. The computational efficiency of the method mostly depends on the efficiency of the community detection algorithm used.

#### Graphlet-based methods

**Figure 1:**
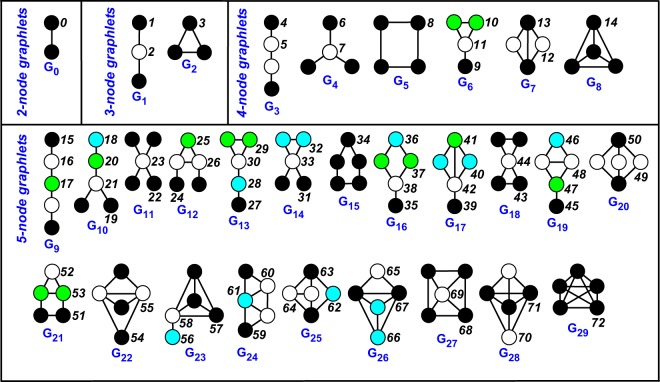
Graphlets (2- to 5-node) in undirected, unweighted networks (from ref. ^22^). The 30 graphlets defined by Pržulj *et al*.^18^ are labeled *G*_0_ to *G*_29_. In each graphlet, nodes with the same shading belong to the same automorphism orbit *O*_0_ to *O*_72_, i.e., they have the same characteristics and are indistinguishable to each other^22^.

Graphlets are small, connected, non-isomorphic subgraphs of large networks. Originally proposed for undirected (unweighted) networks^18^, their use has been subsequently extended to directed networks^24,25^. They encode important information about the structure of the network and provide a valuable tool for comparison. The different types of graphlets need to be enumerated, and this can be done in two ways, i.e., by taking into account or not their automorphism orbits^22^, which differentiate the roles of the nodes in each graphlet (see Fig. 1, where graphlets are enumerated from *G*_0_ to *G*_29_ and orbits from *O*_0_ to *O*_72_). Usually graphlets with more than five nodes are not considered, both for computational reasons and due to repetition of smaller graphlets within their structure.

Counting all graphlets of a network is, in principle, a very demanding task: given a graph with *N* nodes and *L* edges, the worst-case running time for counting 2- to *k*-node graphlets (for both the undirected and directed case) with a complete enumeration strategy is *O*(*N*^*k*^): a tighter upper bound gives *O*(*Nk*_*max*_^*k*−1^), where *k*_*max*_ is the maximum node degree of the graph^20,24^. In practice, these pessimistic bounds are never reached: thanks to the sparsity of real-world networks, and exploiting wiser counting strategies, large improvements are possible. Hočevar and Demšar^26^ proposed the ORCA algorithm, based on a particular counting strategy. Its complexity is *O*(*Lk*_*max*_ + *T*_4_) for the enumeration of 2- to 4-node graphlets, and *O*(*Lk*_*max*_^2^ + *T*_5_) for 2- to 5-node graphlets, where *T*_4_ and *T*_5_ are terms which are negligible in most situations. Aparicio, Ribeiro and Silva^25^ proposed another approach based on a particular data structure, the *G-Trie*^27^: it demonstrated higher performances with respect to ORCA, but its theoretical upper bound is not provided.

Graphlet-based network distances are based on graphlet counts, which can be organized in several ways:

- *Relative Graphlets Frequency Distance* (*RGFD*)^18^. The 29 graphlets from 3 to 5 nodes are counted in each network. Then the distance is defined as $d({G}_{1},{G}_{2})={\sum }_{i=1}^{29}\,|{F}_{i}({G}_{1})-{F}_{i}({G}_{2})|$, where *F*_*i*_(⋅) denotes the count of graphlet *i* normalized with respect to the total number of graphlets in the graph.
- *Graphlet Degree Distribution Agreement* (*GDDA*)^22^. The 73 automorphism orbits of graphlets from 2 to 5 nodes are counted in each network *G*. For each orbit *j* = 0, 1, …, 72, the graphlet degree distribution (GDD) *d*_*G*_^*j*^(*k*), which is the number of nodes in *G* touching *k* times that orbit, is computed. This quantity is first scaled as *d*_*G*_^*j*^(*k*)/*k*, and then normalized by the total area *T*_*G*_^*j*^ under the *j*-th GDD, obtaining *N*_*G*_^*j*^(*k*) = (*d*_*G*_^*j*^(*k*)/*k*)/*T*_*G*_^*j*^. Then, the agreement of the *j*-th GDD between networks *G*_1_ and *G*_2_ is defined as
  3 $${A}^{j}({G}_{1},{G}_{2})=1-\sqrt{\frac{1}{2}\mathop{\sum }\limits_{k=1}^{\infty }\,{({N}_{{G}_{1}}^{j}(k)-{N}_{{G}_{2}}^{j}(k))}^{2}},$$
  and the final GDDA distance is taken as the geometric or arithmetic mean of all the 73 agreements *A*^*j*^(*G*_1_, *G*_2_).
- *Graphlets Correlation Distance* (*GCD*): Yaveroglu *et al*.^19^ investigated the dependencies among graphlets and found that some orbits are redundant, i.e., their count can actually be obtained from the counts of other orbits. Discarding the redundant orbits led to the definition of a more sensible and efficient measure. For instance, graphlets up to 4 nodes have 4 redundant orbits, and discarding them reduces the number of orbits to 11 from the original 15. For a given *N*-node network *G*, the *N graphlets degree vectors*^28^, i.e., the count of the considered orbits that each node touches, are appended row by row to form a *N* × 11 matrix. Then, the Spearman’s correlation coefficient is computed between all column pairs, obtaining the 11 × 11 *Graphlet Correlation Matrix GCM*_*G*_. The GCD distance between graphs *G*_1_ and *G*_2_ is finally defined as the Euclidean distance between the upper triangular parts of the matrices *GCM*_*G*1_ and *GCM*_*G*2_. Yaveroglu *et al*. showed that GCD-11 (the distance with non-redundant orbits) outperformed GCD-15 (with redundant orbits), but also GCD-73 and GCD-56, the distances based on 5-node graphlets with or without reduntant orbits, respectively. Also, GCD-11 performed better than other graphlet-based distances in recognizing different network models.
- *NetDis*^29^: It compares graphlet counts in overlapping neighbourhoods of nodes, rather than in the entire network: more specifically, it considers the 2-step ego-network of each node. The rationale comes from the observation that network size and density strongly influence global graphlet counts, and that such effect can be attenuated by restricting to local subnetworks. Graphlet counts (from *G*_0_ to *G*_29_) are computed for each ego-network and then normalized with respect to the expected counts from a null model. Denote by *S*_*w*_(*G*) the sum over all ego-networks of the normalized count of graphlet *w* in graph *G*. Then, for a given size *k* ∈ {3, 4, 5} of the graphlets:
  4 $$net\,{D}_{2}^{S}(k)=\frac{1}{M(k)}\sum _{w\,{\rm{of}}\,{\rm{size}}\,k}\,(\frac{{S}_{w}({G}_{1}){S}_{w}({G}_{2})}{\sqrt{{S}_{w}{({G}_{1})}^{2}+{S}_{w}{({G}_{2})}^{2}}}),$$
  where *M*(*k*) is a normalizing constant forcing *netD*_2_^*S*^(*k*) ∈ [−1, 1]. Finally, the NetDis distance for *k*-node graphlets is defined as
  5 $$net{d}_{2}^{S}(k)=\frac{1}{2}(1-net{D}_{2}^{S}(k))\,,\,k=\mathrm{3,4,5.}$$
  Note that the NetDis measure actually depends on *k*, which is therefore a parameter to be selected. Yaveroglu *et al*.^20^ pointed out a few critical aspects of the method, such as the choice of a null model, the computational efficiency, and the performances, which are overall inferior to those of other graphlet-based distances.
- *GRAFENE*^21^. In this method, the graphlet degree vectors for graphlets *G*_0_ to *G*_29_ are first computed for each node, and then scaled in [0, 1] dividing each component by the total counts of the corresponding graphlet in the whole network. Principal Component Analysis is then performed over the rescaled graphlet degree vectors, and the first *r* components that account for at least 90% of the total variability are kept. The distance between the two networks is defined as 1 − *d*^*cos*^(*R*_1_, *R*_2_), where *d*^*cos*^ is the cosine similarity and *R*_1_, *R*_2_ are the first *r* principal components for the two graphs. The use of PCA is a novel idea within the graphlet-based methods, that improves the quality of results and the computational performances. Tests on synthetic networks showed that GRAFENE performs at least as well as the other alignment-free methods, and outperforms all other methods on real networks^21^.

Multiple approaches^24,25^ have extended the applicability of graphlet-based methods introducing *directed graphlets*, with the aim of comparing directed (yet unweighted) networks. This allowed to define directed versions of a few of the existing distances^24^: the *Directed Relative Frequency Distance* (*DRGFD*), the *Directed Graphlet Degree Distribution Agreement* (*DGDDA*) and the *Directed Graphlets Correlation Distance* (*DGCD*).

#### Alignment-based methods

These methods create a mapping between the nodes of two graphs (*alignment* procedure) trying to maximize an objective function that captures the quality of matching. They originated in computational biology, where a number of algorithms have been proposed (e.g., refs. ^30–32^): most of them, however, rely on the biological information associated to the network and, as such, have not general applicability. The GRAAL (Graph Aligner) family (GRAAL^33^, H-^34^, MI-^11^, C-^35^, and L-GRAAL^36^), on the contrary, performs network alignment based on the network topology (except the most recent L-GRAAL, which can exploit both biology and topology). Given the graphs *G*_1_ = (*V*_1_, *E*_1_) and *G*_2_ = (*V*_2_, *E*_2_) with |*V*_1_| ≤ |*V*_2_|, a node-mapping *f*:*V*_1_ → *V*_2_ is defined, which induces an edge-mapping *g*:*V*_1_ × *V*_1_ → *V*_2_ × *V*_2_ such that *g*(*E*_1_) = {(*f*(*u*), *f*(*v*)): (*u*, *v*) ∈ *E*_1_}. The objective function that measures the quality of the node alignment *f* is then expressed by the *edge correctness*

6 $$EC=\frac{|g({E}_{1})\cap {E}_{2}|}{|{E}_{1}|},$$

i.e., the fraction of edges in *E*_1_ aligned to edges in *E*_2_. Solving the alignment problem boils out to finding *f* that maximizes *EC*. The GRAAL family proposes a number of different approaches: GRAAL and H-GRAAL assign a similarity score to each pair (*u*_1_, *u*_2_) ∈ *V*_1_ × *V*_2_ based on the graphlet degrees (see above) of the two nodes, thus accounting for the similarity of their neighbourhoods. Then for the alignment GRAAL uses a greedy “seed-and-extend” algorithm trying to maximize the aggregate similarity, while H-GRAAL solves the same problem as an optimal assignment (Hungarian algorithm). In MI-GRAAL a confidence score, computed by taking into account various node statistics (e.g., degree, clustering coefficient, etc.) is assigned to each pair (*u*_1_, *u*_2_) ∈ *V*_1_ × *V*_2_, then nodes are aligned starting from the pairs with highest score. The method is customizable, since any node statistics can be used to compute the confidence score–this allows one the comparison of directed and weighted networks. C-GRAAL does not require an explicit node similarity measure (which however can be incorporated, if available) but works on networks topology with an iterative common-neighbors-based algorithm. Finally, L-GRAAL optimizes a novel objective function that takes into account both sequence-based protein conservation and graphlet-based interaction conservation, by using an alignment heuristic based on integer programming and Lagrangian relaxation. The main drawback of these methods is their computational efficiency, which scales at least quadratically with the number of nodes.

#### Spectral methods

Here the rationale is that, since the spectrum of the representation matrix of a network (adjacency or Laplacian matrix) carries information about its structure, comparing spectra provides metrics for comparing networks. Different approaches are used: Wilson and Zhu^37^ proposed to simply take the Euclidean distance between the two spectra, while Gera *et al*.^38^ proposed to take as distance the *p*-value of a nonparametric test assessing whether the two spectra come from the same distribution. Despite the ease of use, spectral methods prove to suffer from many drawbacks, including cospectrality between different graphs, dependence on the matrix representation, and abnormal sensitivity (small changes in the graph’s structure can produce large changes in the spectrum).

#### NetLSD (Network laplacian spectral descriptor)

This method^39^ summarizes the features of the (undirected, unweighted) graph *G* by a vector derived from the solution of the “heat equation” ∂*u*_*t*_/∂*t* = −*Lu*_*t*_, where *u*_*t*_ is an *N*-dimensional vector and *L* = *I* − *D*^−1/2^*AD*^−1/2^ is the normalized Laplacian matrix. This resembles the dynamics of a continuous-time random walker (e.g., ref. ^40^) so that the solution is a pagerank-like centrality^41^. *L* is symmetric and can be written as *L* = ΦΛΦ^T^ via spectral decomposition, hence the closed-form solution is given by the *N* × *N* “heat kernel” matrix

7 $${H}_{t}={e}^{-Lt}=\Phi {e}^{-\Lambda t}{\Phi }^{{\rm{T}}},$$

whose entry (*H*_*t*_)_*ij*_ is the heat transferred from node *i* to *j* at time *t*. NetLSD condenses the graph representation in the *heat trace signature*

8 $$h(G)={\{{h}_{t}\}}_{t > 0}={\rm{trace}}({H}_{t}).$$

The continuous-time function *h*_*t*_ is finally transformed into a finite-dimensional vector by sampling over a suitable time interval, and the distance between two networks *G*_1_, *G*_2_ is taken as the norm of the vector difference between *h*(*G*_1_) and *h*(*G*_2_). The competitive performance of NetLSD is demonstrated in a few machine learning classification tasks. The time complexity is *O*(*N*^3^), if the full eigendecomposition of the Laplacian is carried out. This would limit the scope of applicability to a few thousands nodes: for that, approximation schemes are devised for reconstructing the spectrum after computing only a limited number of eigenvalues. In this way, networks up to 10^6^ nodes can be treated. Although not discussed in the paper, the method is readily applicable to weighted graphs, whereas the extension to directed networks appears non trivial due to the different spectral characteristics.

#### Portrait divergence

It is a recent method^42^ based on a graph invariant which encodes the distribution of the shortest-path lengths in a graph: the *network portrait*^43^ is a matrix *B* whose entry *B*_*lk*_, *l* = 0, 1, …, *d* (*d* is the graph diameter), *k* = 0, 1, …, *N* − 1, is the number of nodes having *k* nodes at shortest-path distance *l*. The definition also extends to directed and weighted networks–in the weighted case, a binning strategy is needed to manage real-valued path lengths. The network portrait is a powerful summary of the topological features of the graph–e.g., the number of nodes and edges, the degree distribution, the distribution of the next-nearest neighbours, and the number of shortest paths of length *l* can straightforwardly be recovered from *B*. The Portrait Divergence distance between graphs *G*_1_ and *G*_2_ is then defined as follows. First, the probability *P*(*k*, *l*) (and similarly *Q*(*k*, *l*) for the second graph) of randomly choosing two nodes at distance *l* and, for one of the two nodes, to have *k* nodes at distance *l*, is computed:

9 $$P(k,l)=P(k|l)P(l)=\frac{1}{N}{B}_{lk}\frac{1}{\sum _{c}{n}_{c}^{2}}\mathop{\sum }\limits_{k^{\prime} \mathrm{=0}}^{N}\,k^{\prime} {B}_{lk^{\prime} },$$

where *n*_*c*_ is the number of nodes in the connected component *c*. Then, the Portrait Divergence distance is defined using the Jensen-Shannon divergence:

10 $$D({G}_{1},{G}_{2})=\frac{1}{2}KL(P||M)+\frac{1}{2}KL(Q||M)\,,$$

where *M* = (*P* + *Q*)/2 is the mixture distribution of *P* and *Q*, and *KL*(⋅||⋅) is the Kullback-Liebler divergence. The method is computationally efficient for small and medium size graphs, since it is quadratic in the number of nodes, and can naturally handle disconnected networks.

#### Graph kernels

A graph kernel *k*(*G*_1_, *G*_2_) is a non-negative function of two feature vectors *f*_1_, *f*_2_, respectively representative of the two graphs *G*_1_, *G*_2_. In abstract form, a graph kernel implements a (generalized) inner product of the two graphs, which is taken as a measure of their similarity. The proposal of using kernel methods for graph comparison is attributed to refs. ^44,45^ - see also refs. ^46,47^ for a unified description and survey.

The approach is very general, as feature vectors can be defined in very different forms. A recent paper^48^, introducing an R/Python implementation, summarizes 14 different kernel types among the most popular ones: the majority of them are based, in different forms, on statistics on node/edge labels (thus they fall out of the scope of our work, as we do not assume labels on nodes/edges). Two of them are based on graphlet count, and the remaining on the comparison of random walks on the two graphs. But this list is by no means exhaustive, as many other proposals are found in the literature (e.g., ref. ^49^ defines a Laplacian graph kernel). Therefore, graph kernel methods can be considered as a general framework where diversified network features can be included. It follows that selecting the proper graph kernel for the problem at hand can be critical, also considering the non trivial computational requirements of this class of methods: we refer the reader to the discussion in ref. ^47^.

#### Bayes’ modeling of a network population

The network comparison problem can be addressed using a Bayesian nonparametric approach. Durante *et al*.^50^ proposed a mixture model to describe a population of networks, interpreted as realizations of a network-valued random variable, and in particular to infer the parameters of the probability mass function of such variable. This is not a distance-based method, since no explicit distance is defined to compare networks. Instead, the use of a Dirichlet process prior naturally yields a clustering of the input networks thanks to the discreteness of the resulting measure.

#### Persistent homology

Homology is an algebraic-topological measurement of the structure of an undirected unweighted network which, based on the number and dimension of cliques and cycles, exploits the information carried by the mesoscale structure of the network. The generalization to weighted graphs is possible by considering *persistent homology*, which tracks the evolution of cycles when a sequence of unweighted graphs is obtained by thresholding the network at distinct edge weights. This technique was used by Sizemore *et al*.^51^, where suitable quantities derived from persistent homology are jointly used as features to classify networks via hierarchical clustering, showing a superior performance with respect to using standard graph statistics. In the paper, however, no network distance is explicitly defined.

## Results

In this section, we select some of the previously described methods and we define and carry out tests on synthetic networks to assess the performance of each method. Finally we illustrate the application to real-world network data. As a general rule, we only consider methods for which a source/executable code is freely made available by the authors of the method (details of the used codes are in the Supplementary Information file, Sec. S2), in order to fairly test each method in terms of correctness (exceptions are the baseline and spectral methods, requiring the computations of simple distances, which were directly coded by ourselves).

More specifically, among the KNC methods we included:

- The difference of the adjacency matrices (with Euclidean, Manhattan, Canberra and Jaccard norms), as a baseline approach;
- DeltaCon, due to its desirable properties above described, and with the aim of testing a non-trivial KNC method. We also tested the implementation for directed networks.

Among the UNC methods, we selected:

- The (absolute) difference of the clustering coefficient and of the diameter, as baseline approaches;
- For alignment-based methods, MI-GRAAL, which allows to extract additional information from the node mapping;
- For graphlet-based methods, GCD-11 and DCGD-129. The first one is for undirected networks and it was proved to be very effective in discriminating synthetic networks of different topologies^19^. The second one is the directed version of GCD-11, except that, differently from GCD-11, DGCD-129 does not exclude redundant orbits;
- For spectral methods, the Wilson and Zhu^37^ approach: we define three distances by computing the Euclidean distance between the spectra of the adjacency matrices, Laplacians, and Symmetric Normalized Laplacians^52^ (SNLs) (the approach by Gera *et al*.^38^ was discarded since no code was available).
- NetLSD, based on the solution of a dynamical (“heat”) equation;
- Finally, Portrait Divergence, which is naturally able to deal with undirected and directed networks.

No source/executable code was available for Cut Distance and Persistent Homology. The Bayesian method was excluded since no direct comparison is possible with the other approaches, due to its inherently different nature. Finally, GRAFENE was excluded because, despite it is defined for general networks, the executable provided by the authors is strongly domain-dependent, i.e., it requires a biologically-based network definition.

We summarize in Table 1 the methods selected for testing, classified with respect to the type of network they can manage and to the nature of the method itself. In the UNC column, we generically insert “Global statistics” in all rows, although the statistics to be concretely used might depend on the network type. We point out that the tests we carried out on synthetic networks are restricted to the unweighted case: the definition of suitable weighted benchmark networks and of adequate testing strategies for them is a complex and delicate task that goes beyond the scope of the present work. Note that, in the unweighted (binary) case, the MAN and CAN distances actually yield the same result as the (square of the) EUC distance; thus the three distances are actually the same and only the EUC distance will be considered in the analysis.

**Table 1:** Classification of network distances.

Network type	Known Node-Correspondence (KNC)	Unknown Node-Correspondence (UNC)
Undirected Unweighted	–Euclidean (EUC), Manhattan (MAN), Canberra (CAN), Jaccard (JAC) distances –DeltaCon (DCON)	–Global statistics –Spectral Adjacency (EIG-ADJ), Laplacian (EIG-LAP), SNL (EIG-SNL) distances –GCD-11 –MI-GRAAL –NetLSD –Portrait Divergence (PDIV)
Directed Unweighted	–Euclidean, Manhattan, Canberra, Jaccard distances –DeltaCon	–Global statistics –DGCD-129 –MI-GRAAL –Portrait Divergence
Undirected Weighted	–Euclidean, Manhattan, Canberra distances –Weighted Jaccard distance (WJAC)	–Global statistics –Spectral Adjacency, Laplacian, SNL distances –MI-GRAAL –NetLSD –Portrait Divergence
Directed Weighted	–Euclidean, Manhattan, Canberra distances –Weighted Jaccard distance	–Global statistics –MI-GRAAL –Portrait Divergence

### Perturbation tests

We perform successive perturbations starting from an original graph and we measure, after each perturbation, the distance of the obtained graph from the original one. This is aimed at checking to what extent the considered network distance has a “regular” behaviour. As a matter of fact, we expect that whatever distance we use, it should tend to zero when the perturbations tend to zero, i.e., the distance between very similar graphs is small; moreover, the distance should increase monotonically with the number of perturbations, meaning that fluctuations, if any, should be very limited. In addition, for perturbations that tend to randomize the graph (see below), the distance should saturate to some asymptotic value after a large number of perturbations, because when the graph has become fully randomized it remains such for any further perturbation (see ref. ^16^ for a formal discussion on the properties of similarity measures).

The following types of perturbations are considered:

- *Removal* (*pREM-test*): a connected node pair is picked uniformly at random and the edge is removed (the graph density decreases).
- *Addition* (*pADD-test*): a non-connected node pair is picked uniformly at random and an edge is added (the graph density increases).
- *Random switching* (*pRSW-test*): combines the previous two: a connected node pair is picked uniformly at random and the edge is removed; then a non-connected node pair is picked uniformly at random and an edge is added (the graph density does not change).
- *Degree-preserving switching* (*pDSW-test*): two edges are picked uniformly at random and swapped: if we pick (*i*, *j*) and (*u*, *v*), we delete them and insert the new edges (*i*, *v*) and (*j*, *u*), provided they are not already existing (the graph density and the degree distribution do not change).
  In addition, for directed networks:
- *Change of direction* (*pDIR-test*): a connected node pair is picked uniformly at random and the edge direction is reversed (the graph density does not change).

We consider two families of networks, i.e., undirected and directed, and three models for each family, i.e., Erdös-Rényi (ER)^53^, Barabási-Albert (BA)^54^ and Lancichinetti-Fortunato-Radicchi (LFR)^55,56^ (see ref. ^16^ for alternative proposals of synthetic test networks for comparison methods). The three models, which are well-known and widely used as test-beds in a variety of network science problems^2^, have increasing structural complexity. In the undirected case, an ER network^53^ is built by setting the number of nodes and links, and then by randomly assigning links to node pairs selected uniformly at random. This yields a network with almost homogenous degree distribution, i.e., all nodes are statistically equivalent and have small fluctuations around the average degree. In a BA network^54^, on the other hand, a few nodes with very high degree coexist with a majority of medium/small degree nodes, giving rise to a strongly inhomogeneous (power-law) degree distribution, as encountered in a number of real-world datasets. LFR networks^55,56^ add a further level of complexity, namely modular structure: the network is partitioned into communities, which are subgraphs with large internal density yet loosely connected to the other communities, and not only the nodes degree, but also the communities size are power-law distributed, to mimic features typically found in real data. The three models, originally developed for undirected networks, have been generalized to the directed case (see Supplementary Information file, Sec. S3, for details on the creation of undirected and directed networks).

For each family/model pair, we create two graphs with 1000 nodes, respectively with density 0.01 and 0.05. On each network, we perform 10 different replications of 1000 successive perturbations of the types above defined. For the undirected and directed cases, respectively, we test the methods in the first and second row of Table 1. For “Global statistics”, we use the clustering coefficient and the diameter for undirected networks, and the diameter only for directed networks. For density 0.05 and for all directed networks, we exclude MI-GRAAL from the analysis because it turns out to be computationally too heavy.

**Figure 2:**
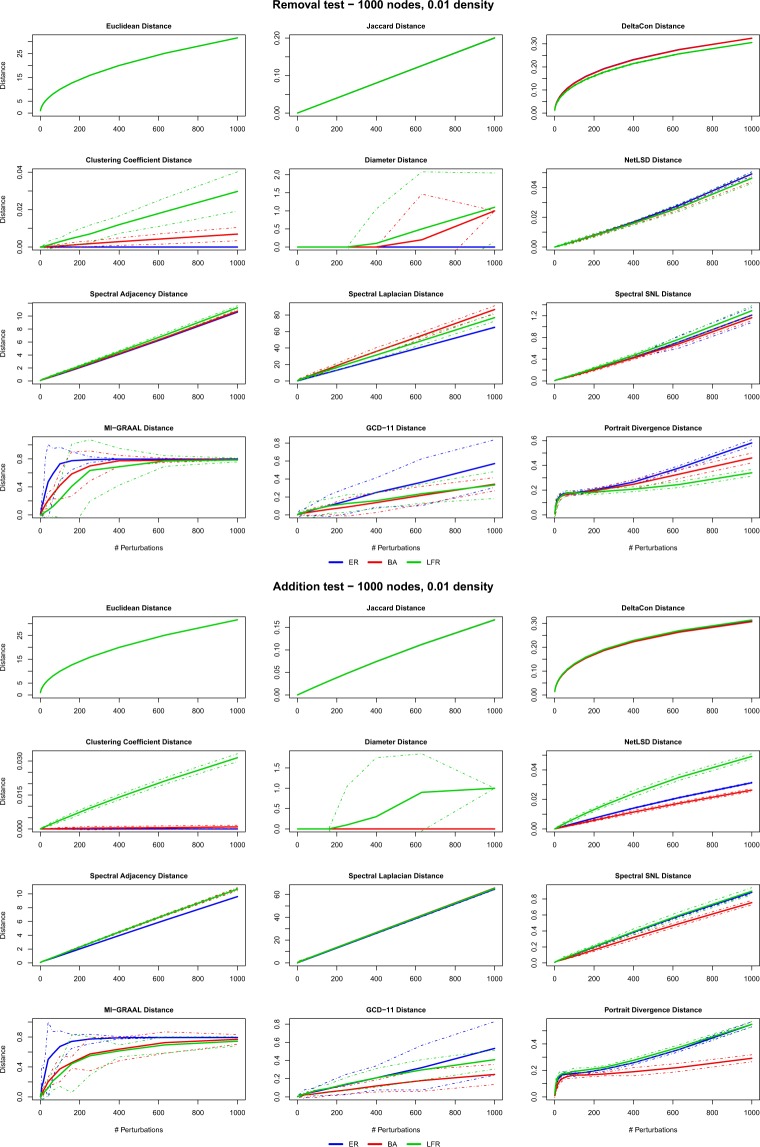
Perturbations tests: results of the *Removal* (*pREM-test*) and *Addition* (*pADD-test*) tests for the 12 distances and the 3 undirected models ER, BA, LFR (density 0.01). Solid (dashed) lines are the mean (±3 std) values obtained over the 10 replications of the perturbation histories.

**Figure 3:**
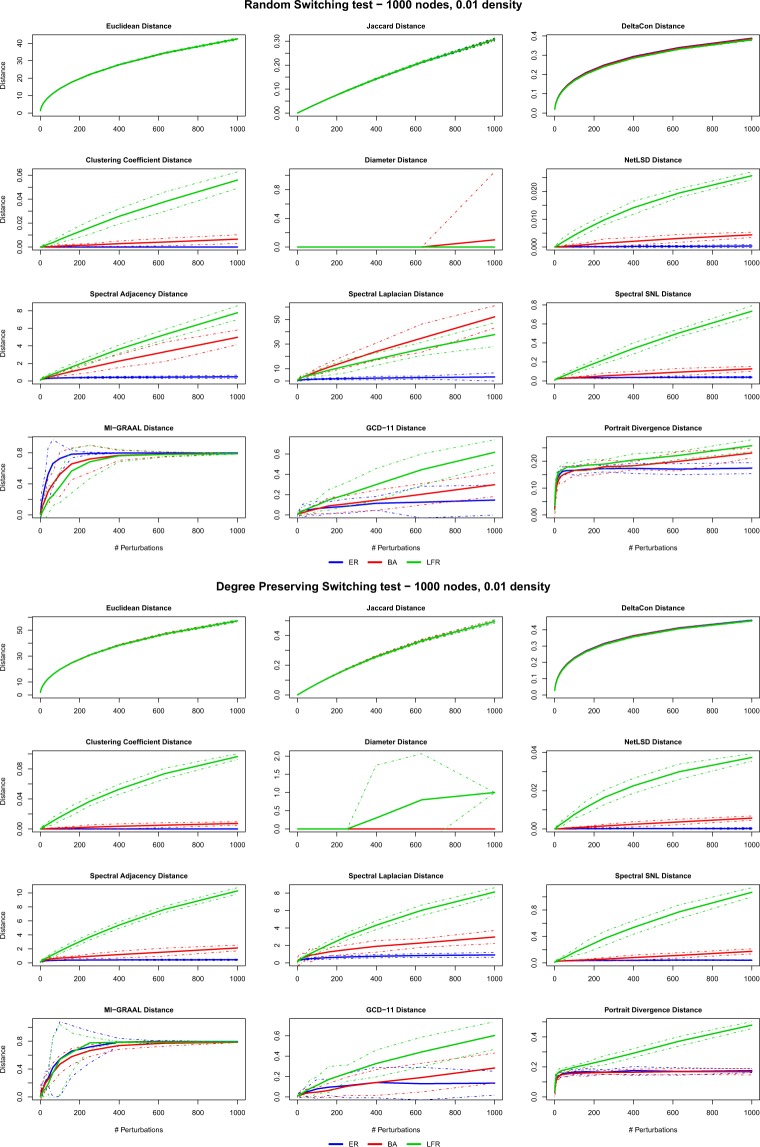
Perturbations tests: results of the *Random switching* (*pRSW-test*) and *Degree-preserving switching* (*pDSW-test*) tests for the 12 distances and the 3 undirected models ER, BA, LFR (density 0.01). Solid (dashed) lines are the mean (±3 std) values obtained over the 10 replications of the perturbation histories.

Figures 2 and 3 summarize the results obtained on undirected networks with density 0.01 (see Supplementary Information file, Figs. S1 and S2, for density 0.05). We notice that essentially all distances have a “regular” behaviour, in the sense above discussed. The only remarkable exception is the diameter distance, which remains zero on a broad range of perturbations for most network models, thus proving inadequate as a network distance. Confidence bands are generally tight, except for MI-GRAAL and GCD-11 which fluctuate more. In a few cases, the distances tend to saturate–this is especially evident in switching tests (*pRSW-test* and *pDSW-test*). Overall, these results denote that all distances are well defined and can be properly used for comparison–except the diameter, which therefore will not be considered in the remainder.

At the same time, the tests reveal a different behaviour for the two classes of distances KNC and UNC (Table 1). KNC distances turn out to be practically insensitive to the network model and even to the type of perturbation: for these distances, a perturbation acts in the same way in all the considered models. This is not unexpected: for example, EUC simply measures the (square root of the) number of edges that have been added, removed or switched, disregarding the topology in which these changes happen: a very weak characterization of the effects of the perturbations.

UNC distances, on the contrary, show quite different values and patterns for different models and perturbations. The three spectral distances behave quite differently from one test to another. The trend is linear in *pADD-test* and *pREM-test* and independent on the network model, while for *pRSW-test* and *pDSW-test* the distance is definitely larger for LFR networks, in almost all cases. This behaviour captures the higher sensitivity of LFR networks to random rewiring, due to the progressive destruction of the built-in community structure via removal of intra-communities edges and creation of inter-community connections. Indeed, the same behaviour is shown by the clustering coefficient and the NetLSD distance. On the other hand, *pRSW-test* affects BA networks too, at least for a few of the distances, because hubs will easily lose connections. Noticeably, ER networks are weakly affected by random rewiring of both types, if measured by spectral distances: random rewiring yields of course different graphs, but structurally equivalent to the original one.

Coming to the last UNC distances, we notice the comparatively large variability of GCD-11. PDIV, on the other hand, has a peculiar behaviour: the distance has a steep increase in the very first perturbations, then either it increases further but at a slower rate, or it immediately saturates. The latter is the case, e.g., of ER and BA models under *pDSW-test*: under random rewiring, the ER topology yields equivalent graphs that are almost equally distant from the original one, but interestingly the same happens for the BA structure: we argue that after some *pDSW-test* steps the shortest paths distribution is almost the same for all the perturbed graphs, so that the PDIV distance becomes constant.

The MI-GRAAL distance shows a behaviour different from all the other distances. The confidence bands reveal a large variability, as above pointed out. In a few extreme cases (e.g., *pREM-test* and *pDSW-test* on LFR graphs, or *pADD-test* and *pDSW-test* on ER graphs), the confidence band spans almost the entire [0, 1] range, namely, a few perturbations may lead to a graph apparently equal or totally different from the original one, depending on which edges are perturbed. This unpleasant behaviour is probably due to the large sensitivity to perturbations of the local (node) features. Recall that MI-GRAAL distance builds a map between the most similar nodes of the compared networks. Perturbations induce differences in the individual node features, i.e., degree, clustering coefficient, and betweenness centrality, that can obscure and shuffle the correct node correspondence. Incidentally, ER networks seem the most suffering ones, presumably because nodes are weakly characterized.

**Figure 4:**
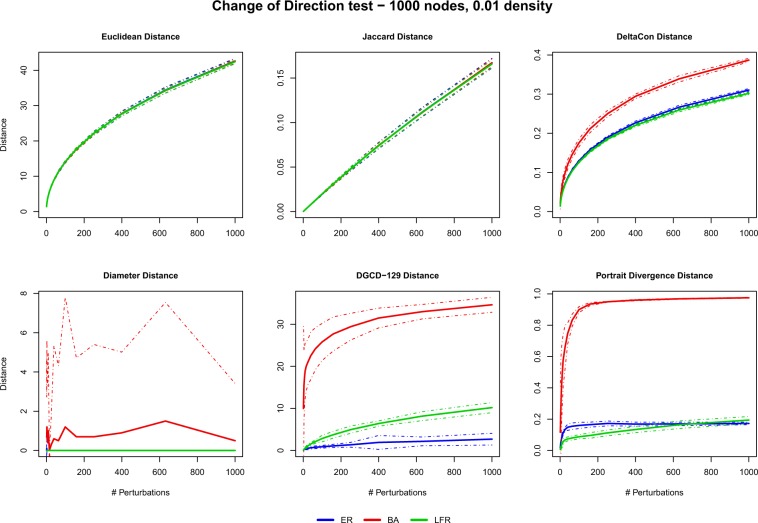
Perturbations tests: results of the *Change of direction* (*pDIR-test*) test for the 6 distances (see Table 1, MI-GRAAL excluded for computational reasons) and the 3 directed models ER, BA, LFR (density 0.01). Solid (dashed) lines are the mean (±3 std) values obtained over the 10 replications of the perturbation histories.

Finally, Fig. 4 summarizes the results of the change of direction (*pDIR-test*) test on directed networks with density 0.01 (see Supplementary Information file, Figs. S3 to S5, for density 0.05). Again, for the same reasons as above, the simplest distances (EUC and JAC) show a behaviour which is very regular and insensitive to the network model. Again the diameter distance has a pathological behaviour and should be discarded in any case. What emerges in the remaining two UNC distances, on the other hand, is their strong sensitivity in the case of BA networks, where (see DGCD-129 and PDIV panels) even the first few perturbations yield large distances. To explain this effect, it should be emphasized that in our directed BA networks, by construction, hubs are nodes having systematically large in-degree while the out-degree is homogeneously distributed (see Supplementary Information file, Sec. S3, for details on the construction of directed networks). It turns out that few changes in the edge directions are sufficient to dramatically change the number and type of graphlets (DGCD-129) and the structure of the shortest paths (PDIV). Such a strong sensitivity could be an issue in some applications, although it is certainly amplified by the specific structure of our benchmark networks.

### Clustering tests

In this section, several tests are proposed aimed at assessing the effectiveness of each method in recognizing and grouping together networks with the same structural features, i.e., originated from the same model. In other words, to yield a good performance, a method should be able to assign small distance to network pairs coming from the same model but large distance to pairs coming from different models. We adopt a typical approach for testing performance of methods operating in a not-supervised setting, i.e., simulating controlled scenarios where groups (or, as in this case, network models) are known, then running the algorithms as in the unsupervised case, and finally checking their ability in properly identifying groups coming from different models. Note that only UNC methods are suitable to this task, since we assume that no node correspondence is available. Thus, with reference to Table 1, we test the methods of the UNC column, in the first and second row, respectively, for undirected and directed networks. In both cases we exclude MI-GRAAL, which proved to be computationally too heavy in most of the experimental settings below described. We also restrict the analysis of “Global statistics” to the clustering coefficient (only for undirected networks), given the bad performance of the diameter in the perturbation tests above described.

We choose again ER, BA, and LFR network models. For each model, we consider two sizes (1000 and 2000 nodes), two densities (0.01 and 0.05), and the two families undirected/directed (see Supplementary Information file, Sec. S3, for details on network construction), and we generate 5 networks for each one of the 3 × 2 × 2 model/parameter set, for a total of 60 undirected and 60 directed networks. We then compute all the pairwise distances for each method, ending up with two (i.e., undirected/directed) 60 × 60 distance matrices.

**Figure 5:**
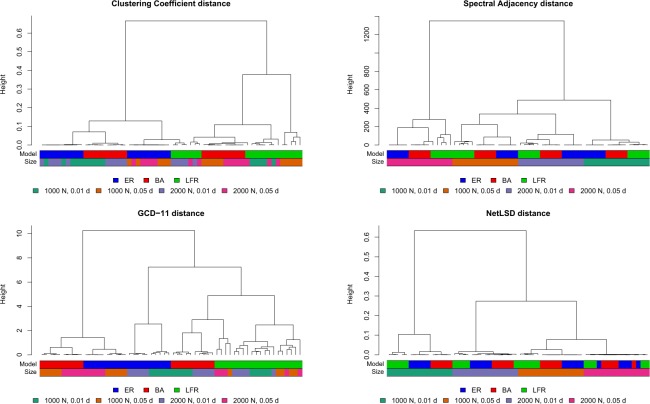
Clustering tests: dendrograms for the undirected case (the dendrograms EIG-LAP, EIG-SNL, and PDIV are similar to EIG-ADJ and are reported in the Supplementary Information file, Fig. S6).

**Figure 6:**
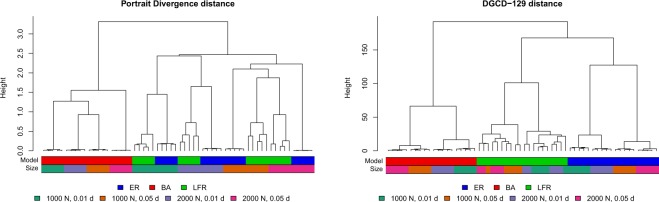
Clustering tests: dendrograms for the directed case. Left: Portrait Divergence distance PDIV. Right: DGCD-129 distance.

In Figs. 5 and 6 we visualize the results by means of dendrograms (built with *ward.D2* linkage^57^ and Optimal Leaf Ordering^58^) where, to aid interpretation, we label each leaf (=network) with two colours, one denoting the network model and the other the size/density. This is in line with the aforementioned purpose: since we can only control the network generating mechanisms, and not the realizations obtained by each of them, we simulate a setting as controlled as possible, mimicking a supervised context, and then we assess the performances of the different methods when adopted for building clusters, as in the unsupervised setting. Thus the supervised context is used only to set the benchmarking object, then the algorithms run in a completely unsupervised way. For this reason, as above emphasized, it is reasonable to expect that an effective method should ideally be able to cluster all networks coming from the same model family together, without being disturbed from the different sizes and densities.

In the undirected case (Fig. 5), we observe that all methods are able, more or less precisely, to correctly group networks of the same class, but only if they have the same size/density. This is the case for clustering coefficient distance, EIG-ADJ (as well as EIG-LAP, EIG-SNL and PDIV, not reported in the figure), and NetLSD. GCD-11 achieves a definitely better performance, as it proves able to group all LFR networks, along with the BA graphs with low densities, and by identifying two other clear clusters: one containing ER graphs with low density, and the other BA and ER graphs with high densities. Overall, the results reveal a quite strong dependence of all methods, except GCD-11, on size and density of the graphs, a result not fully satisfactory.

In the directed case (Fig. 6), PDIV behaves better than in the undirected case, since it is able to group together all the BA graphs, while ER and LFR graphs are grouped together in another large cluster within which density becomes the discriminating factor. On the other hand, DGCD-129 is able to achieve a perfect grouping of the network models.

**Figure 7:**
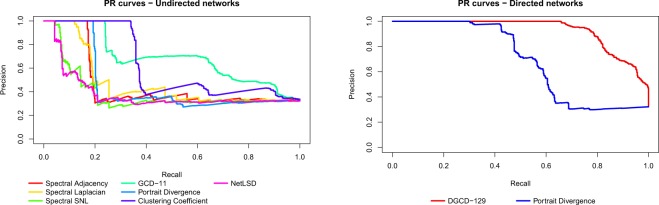
Clustering tests. Precision-Recall curves for the methods used for undirected (left) and directed (right) networks.

A systematic approach to comparatively quantify the performance of different methods is the Precision-Recall framework^19,20,24^: for a given network distance, one defines a threshold *ε* > 0 and classifies two networks as belonging to the same model class if their distance is less than *ε*. Given that the correct classes are known, the accuracy of classifying all network pairs can be quantified by Precision and Recall. The procedure is then repeated by varying *ε*, obtaining the Precision-Recall curve (Fig. 7) which, ideally, should have Precision equal to 1 for any Recall value. Overall, the curves highlight the superiority of the graphlet-based approaches (GCD-11 and DGCD-129, respectively) in comparison to all other methods we have tested–their curve is above all the others in almost the entire curve range.

A quantification of the performance of each method can be obtained by the Area Under the Precision Recall curve (AUPR), which should be 1 in the ideal case. For the left panel of Fig. 7, AUPR ranges from 0.386 (NetLSD) to 0.688 (GCD-11), for the right panel AUPR is 0.685 for PDIV and 0.928 for DGCD-129. We note that all methods perform better than a random classifier (AUPR equal to 0.322), but the improvement is very moderate for spectral methods and NetLSD. The performance of the clustering coefficient distance (undirected networks) is surprisingly good, with AUPR equal to 0.620. At least in our testing environment, this simple statistics appears to be a viable low-cost alternative to more sophisticated distances.

In addition, the AUPR measure can be used to confirm that, if only networks of the same size/density are considered, all the analysed methods yield an essentially correct classification–a feature already highlighted above when discussing the dendrograms. Indeed, we report that in this case almost all methods of Fig. 7 obtain an AUPR larger than 0.8 in the undirected case, and practically equal to 1 in the directed case (see Supplementary Information file, Tables S3 and S4).

### Tests on real-world networks

In this section we assess the performance of the above methods in grouping sets of real-world networks: our aim is to understand more deeply how these methods work and which type of results one should expect from their use. We analyse multiplex networks, since their layers can be seen as individual (ordinary) networks defined over the same node set. This is a desirable property for our analysis, because it enables the use of both KNC and UNC methods. One can therefore ascertain whether the two classes of methods provide different insights of the same problem.

#### European air transportation network

This network has 448 nodes, representing European airports, and 37 layers, corresponding to the airline companies (see Supplementary Information file, Table S5, for the list), for a total of 3588 undirected and unweighted edges^59^. We test all the distances of the first row of Table 1, namely three KNC distances (recall that MAN and CAN are equivalent to EUC in this setting) and eight UNC distances (with the clustering coefficient as “Global statistics”).

**Figure 8:**
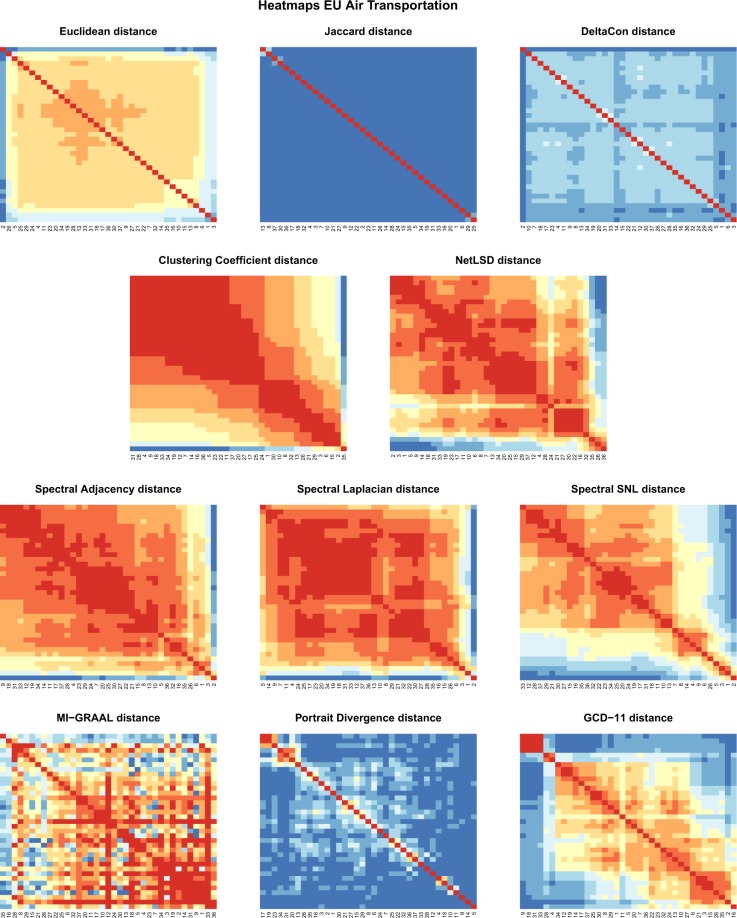
European Air Transportation Network: Distance matrices for three KNC distances (first row) and eight UNC distances (second to last row). The colour in entry (*i*, *j*) is the distance between layers *i* and *j* of the multiplex air transportation network. Distances are coded by colours from blue (large distance) to red (small distance).

Figure 8 displays the eleven 37 × 37 (symmetric) distance matrices (the corresponding dendrograms are in the Supplementary Information file, Fig. S7). We firstly observe that the two classes of distances yield qualitatively different output: KNC distances are almost uniform over the entire matrix (JAC above all), while UNC methods obtain different degrees of differentiation, remarkably for EIG-SNL and GCD-11. EUC and DCON essentially identify one single (trivial) cluster including all the networks except Ryanair (#2). This is not unexpected, since the Ryanair layer differs from the others in having by far the largest number of edges and (non isolated) nodes. In turn, this demonstrates that KNC distances are strongly affected by size and density: two graphs with different sizes or densities are classified as distant just because of edge counting, regardless of their topological properties.

The three spectral distances have similar behaviour. Dendrograms identify two main clusters, a small one containing airlines #1, #2, #3, and, according to the method, #5, #6 or #26, and a large one containing all the rest (actually EIG-SNL reveals a third, small cluster, which however has no clear interpretation). The small cluster groups together the airlines with the highest number of nodes and connections (>75 nodes, >110 edges). This confirms the strong dependence of the three spectral distances on size and density, as highlighted in the synthetic tests.

To avoid ambiguities in the interpretation of the heatmaps of Fig. 8, we remark that the presence of a group of airlines with comparatively smaller internal distance (i.e., a diagonal block with hotter colour) does not imply the existence of a well defined group, unless the group itself displays comparatively higher distance (colder colour) to the remaining airlines. That is why, for example, in the EUC case airlines #34, #19, #12, etc., do not form a well separated group, as well as the hotter coloured diagonal blocks in the EIG-ADJ case are not significant since there is no sharp colour separation (i.e., cold colour outside the block) but a continuum of distances among airlines.

The patterns of the clustering coefficient and of the NetLSD distance matrices are qualitatively similar to that of the spectral distances. NetLSD matrix does not lend to a clear interpretation. The clustering coefficient matrix is easy to read, on the contrary, thanks to the straightforward definition of the specific network distance: airline #35 (Wideore) has by far the largest clustering coefficient. In addition, the heatmap reveals two weakly separated groups, which indeed correspond to the groups of airlines with, respectively, larger (>0.1) and smaller clustering coefficient.

MI-GRAAL and PDIV distances do not show any meaningful grouping of networks, except that the former groups together three pure-star networks (#9, #31, #33) but misses the fourth one existing in the database (#18), and the latter groups a few networks having the same maximum degree (#17, #19, #23, and #13, #20, #31, #34) but in fact misses a few others. Finally, the distance matrix and the dendrogram for GCD-11 show three main clusters. One of them contains all the four pure-star layers in the dataset (#9, #18, #31, #33), while the second groups seven of the eight airlines with comparatively large (>0.15) clustering coefficient (#2, #3, #6, #15, #16, #21, #26, #35). The third cluster contains all the remaining layers. In addition, airlines #4 and #28 are also paired to form a small but well identified cluster: it turns out that they both have zero clustering coefficient without being a star graph. Overall, these results denote that GCD-11 is quite effective in classifying networks based on purely topological features.

**Figure 9:**
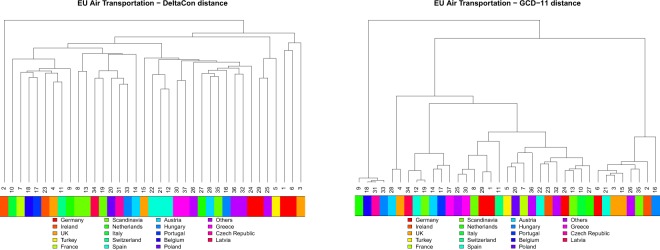
European Air Transportation Network: dendrograms for DCON and GCD-11, with airlines coloured according to the nations they are based in. Contrarily to UNC distances (e.g. GCD-11), KNC distances (e.g., DCON) are able in many cases to group airlines which are based in the same nation.

We pointed out above that KNC distances are unable to extract meaningful clusters of networks. Nonetheless they provide some useful result, as they pair networks on a regional basis, i.e., they put at a comparatively small distance airlines based in the same nation/region. For example, EUC dendrogram pairs #1 with #6 (Germany), #8 with #13 (Scandinavia), #30 with #37 (Greece), and #9 with #27 (Netherlands). JAC pairs together the aforementioned airlines and, in addition, #3 with #4 (UK), #12 with #22 (Spain), #2 with #23 (Ireland), and #16 with #33 (Hungary). DCON (see Fig. 9) groups #8 with #13 (Scandinavia), #12, #21, #22 (Spain), #30 with #37 (Greece), #1 with #6 (Germany), and #24 with #29 (Germany again). This kind of grouping is not recovered by UNC distances (see, e.g., DCON vs GCD-11 in Fig. 9). As a matter of fact, KNC distances are all based on some sort of node similarity. Airlines based in the same nation share the same national airports; moreover, in many instances the two airlines paired by KNC methods are the leading national company and a low-cost company, offering different journey and price conditions on the same routes. Consequently the nodes of two airlines based in the same nation are typically similar and this reduces the distance between the corresponding networks.

#### FAO trade network

We now consider the FAO Trade Network of food, agricultural and animal products^60^. It is composed of 364 layers, each one representing a different product, sharing 214 nodes, corresponding to countries, for a total number of 318346 connections. If layers/products are matched with the WTO Harmonized System classification^61^, we find that FAO products belong to seven out of the fifteen major HS categories: *Animals and Animal Products*; *Vegetable Products*; *Foodstuffs*; *Chemicals*; *Plastic and Rubbers*; *Raw Hides, Skins and Leathers*; *Textiles*. Each layer is directed and weighted, the weights representing the export value (thousands of USD) of a product from one country to another. Here we discuss a few results derived on the binarized version of the network, which is obtained, for each product *p*, by retaining only the links departing from countries *c* having Revealed Comparative Advantage *RCA*_*cp*_ > 1, i.e., countries which are significant exporters of that product (see refs. ^62,63^. and Supplementary Information file, eq. (S1), for details). We test the distances of the second row of Table 1, namely three KNC distances (since MAN and CAN are equivalent to EUC) and two of the UNC distances (as usual, we exclude MI-GRAAL due to the high computational cost, and we do not consider the clustering coefficient since we analysed it as “Global statistics” for undirected networks only).

**Figure 10:**
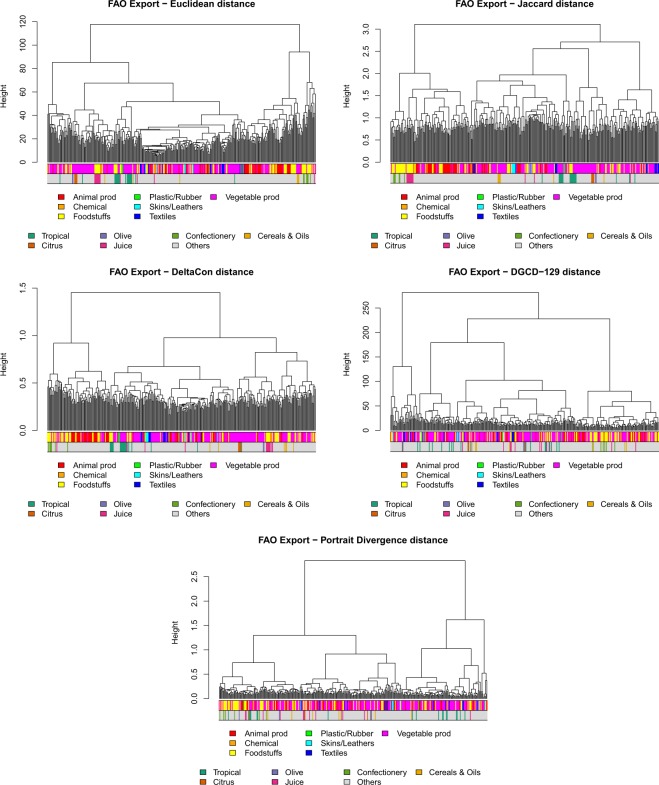
FAO Trade Network: cluster analysis for the methods under test. In the upper colour bar, products are classified according to WTO Harmonized System. In the bottom bar, a few specific categories are evidenced.

The cluster analysis summarized by the dendrograms of Fig. 10 clearly shows that the naive expectation that products organize in groups according to their category is not fulfilled: HS categories turn out to be too broad and diversified to induce similar trade topologies. *Vegetable Products*, for example, is the most represented category: it includes such a diversity of products that the corresponding networks are also extremely diverse, not only in terms of number and location of producing/consuming countries, but also for the possible existence of intermediate countries where products are imported, transformed, and eventually re-exported.

Nonetheless, cluster analysis is able to spot interesting similarities. For example, some agricultural products (such as tropical fruits or citrus fruits) can be produced only in specific regions of the world and thus only by few countries. Thus, we expect that the distance between the corresponding layers will be small–this obviously holds true if KNC distances are used. This expectation is indeed confirmed. All KNC distances group together (see the bottom colour bars in Fig. 10) all the citrus fruits (namely *Oranges*; *Lemons and limes*; *Grapefruit*; *Tangerines, mandarins, clementines, satsumas*) present in the dataset, and EUC also puts *Olives preserved* and *Oil, olive, virgin* close to them. This is not surprising, since the latter are mostly produced in the same areas as citrus, for climatic reasons. Most of the tropical fruits are also gathered together: EUC mainly finds two groups, one which contains *Cocoa, beans*; *Cocoa, powder and cake*; *Cocoa, paste*; *Cocoa, butter*; *Bananas*; *Pineapples*; *Mangoes, mangosteens, guavas*; and the other one which contains *Vanilla*; *Coconuts*; *Coconuts, desiccated*; *Cinnamon*; *Nutmeg*; *Pepper*; *Coffee, green*. Similarly, DCON also identifies two major groups of tropical fruits. In addition, another group of specific products, namely those related to juices, are grouped together by KNC distances. Notably, the groups of citrus fruits and citrus juices are not adjacent to each other, revealing that production and processing of fresh fruit mostly take place in different countries thus yielding different trade networks.

Finally, UNC distances, applied to the FAO dataset, identify those few products with a very peculiar trade pattern. For example, the leftmost leaves of the DGCD-129 dendrogram (Fig. 10), which have large distance from all other leaves, correspond to trade networks with a pronounced star-like structure. They relate to very peculiar products, such as *Bulgur*; *Maple sugar and syrups*; *Margarine, liquid*; *Beehives*; *Camels*; and others. Some of them are produced and exported by only one country (e.g., *Maple sugar and syrup*) or are imported significantly by very few countries (e.g., *Beehives*) and thus their trade patterns are highly centralized.

## Discussion and Concluding Remarks

We reviewed a broad set of methods available in the literature for network comparison, assessing their performances both on synthetic benchmarks and on real-world multilayer networks. We relied on a well-accepted dichotomy of the methods based on their functioning. The first class (Known Node-Correspondence) gathers all the methods, such as Euclidean, Jaccard or DeltaCon distances, which require a priori to know the correspondence between the nodes of the compared networks: they are suitable to quantify the differences between graphs representing various connection patterns among the same node set, as in the EU Air Transportation case, or to quantify changes or spotting anomalies occurring in a graph subject to temporal evolution. The second class (Unknown Node-Correspondence) collects all the methods which do not require any a priori knowledge of the correspondence between nodes. Methods of this class, such as spectral distances, graphlet-based measures, and Portrait Divergence, are specifically suited for structural comparison, namely to provide information about how much, and in what sense, the structures of graphs differ.

To evaluate the performance of each method, we carried out two classes of tests–perturbation and clustering–with the use of synthetic networks. Virtually all methods demonstrated a fairly good behaviour under perturbation tests (the diameter distance being the only exception), in the sense that all distances tend to zero as the similarity of the networks increases, tend to a plateau after a large number of perturbations, and do not fluctuate too much if perturbations are repeated. On the contrary, the clustering tests highlighted different behaviours and performances. When networks of the same size and density are considered, in both the undirected and directed case most methods are reasonably able to discriminate between different structures, often achieving a perfect classification. However, when considering networks of different sizes and densities, the results change considerably. In the undirected case, the graphlet-based measure GCD-11 is the best performing distance in discriminating between different network topologies and clearly outperforms all the other methods whereas, on the other hand, a few methods only perform slightly better than a random classifier. In the directed case, the graphlet-based measure DGCD-129 is able to achieve an almost perfect classification, whereas, for example, Portrait Divergence distance performs much better than in the undirected case. Therefore, since in many real-world applications density and size of the graphs may vary considerably, graphlet-based measures prove to be the most reliable tools to investigate the differences between networks structures. As a matter of fact, while in the perturbation tests graphlet methods display behaviours comparable to the other methods in terms of regularity, in clustering tests GCD-11 and DGCD-129 demonstrate performances overall superior to the other methods. This emerges from the dendrograms (Figs. 5 and 6) and from the Precision/Recall diagrams (Fig. 7), the latter synthesized by the AUPR values (see Supplementary Information file, Tables S3 and S4). Needless to say, these conclusions are related to our specific test-bed, and we cannot exclude that different evidences could emerge from the analysis, e.g., of different network topologies, where some other method could prove to be more effective.

The methods analysed were able to recover important features in the real-world case studies analysed. For example, in the European Air Transportation Network they paired at small distances airlines based in the same nation, which are expected to have high nodes similarities since they share the same airports and routes. In the FAO Trade Network, they were able to group specific products whose countries of production are in common due to climate reasons. In this latter case, results were obtained after the original weighted network was reduced to a binary (unweighted) version. In this respect, considerable work is still needed to develop and systematically test methods able to fully exploit the available information on weights, an effort that could potentially yield a significant improvement in network classification. A common feature of methods with Known Node-Correspondence, that emerged from the analysis of real-world case studies, is their strong dependence on density and size of the graphs–a feature that could be a strength or a weakness according to the application. More in general, the analysis of real-world datasets has shown that, even when a network measure is unable to induce a clear partition of the entire set of networks, it can nonetheless highlight important subsets. In this sense, using different methods–based on different notions of network similarity–can provide rich and diversified information.

## Supplementary information

Supplementary Information
