# Supplementary material for: Comparing methods for comparing networks
Source: Sci Rep. 2019 Nov 26;9:17557. doi: 10.1038/s41598-019-53708-y (PMC6879644; doi:10.1038/s41598-019-53708-y)
Supplement: Supplementary file 1 — Supplementary Information [file 41598_2019_53708_MOESM1_ESM.pdf]

Supplementary Information
Comparing methods for comparing networks

Mattia Tantardini[1], Francesca Ieva[2,3], Lucia Tajoli[4], and Carlo Piccardi[5,*]

[1]Moxoff SpA, via Schiaffino 11/A, 20158 Milano, Italy
[2]MOX - Modelling and Scientific Computing Lab, Department of Mathematics, Politecnico di Milano, Via Bonardi 9, 20133 Milano, Italy
[3]CADS - Center for Analysis, Decisions and Society, Human Technopole, 20157 Milano, Italy
[4]Department of Management, Economics and Industrial Engineering, Politecnico di Milano, Via Lambruschini 4/b, 20156 Milano, Italy
[5]Department of Electronics, Information and Bioengineering, Politecnico di Milano, Piazza Leonardo da Vinci 32, 20133 Milano, Italy
[*]carlo.piccardi@polimi.it

S1 Norm of the difference of the adjacency matrices

We specify the details of the definition of the four matrix norms used as network distances in the paper. Given two networks $G_1(V_1,E_1)$ and $G_2(V_2,E_2)$ with adjacency matrices $A_1 = [a^1_{ij}]$ and $A_2 = [a^2_{ij}]$, respectively, and identical node sets $V = V_1 = V_2$ (if $V_1 \neq V_2$, we take $V = V_1 \cup V_2$ and pad with zeros the adjacency matrices), we define:

– *Euclidean distance (EUC):* $d_{EUC}(G_1,G_2) = \sqrt{\sum_{i,j\in V}\left(a^1_{ij} - a^2_{ij}\right)^2}$

– *Manhattan distance (MAN):* $d_{MAN}(G_1,G_2) = \sum_{i,j\in V} |a^1_{ij} - a^2_{ij}|$

– *Canberra distance (CAN):* $d_{CAN}(G_1,G_2) = \sum_{i,j\in V} \frac{|a^1_{ij}-a^2_{ij}|}{|a^1_{ij}|+|a^2_{ij}|}$, where we set $|a^1_{ij}| + |a^2_{ij}| = 1$ if $a^1_{ij} = a^2_{ij} = 0$

– *Jaccard distance (JAC):* $d_{JAC}(G_1,G_2) = 1 - J(A_1,A_2) = 1 - \frac{|A_1\cap A_2|}{|A_1\cup A_2|}$, where $J(A_1,A_2)$ is the Jaccard similarity, and union and intersection are meant elementwise. The Jaccard distance can be generalized to handle weighted networks, both directed and undirected: the Weighted Jaccard similarity[1,2] is defined as:

$$J_W(A_1,A_2) = \begin{cases} \dfrac{\sum_{i,j\in V} \min(a^1_{ij},a^2_{ij})}{\sum_{i,j\in V} \max(a^1_{ij},a^2_{ij})} & \text{if } \sum_{i,j\in V} \max(a^1_{ij},a^2_{ij}) > 0 \\ 1 & \text{if } \sum_{i,j\in V} \max(a^1_{ij},a^2_{ij}) = 0 \end{cases}$$

and the Weighted Jaccard distance (WJAC) as

$$d_{WJAC}(G_1,G_2) = 1 - J_W(A_1,A_2)\,.$$

S2 Codes/executables used for testing

The codes/executables used in the analysis have been downloaded from the following URLs (the norms of the differences between adjacency matrices, the global statistics, and the spectral methods were straightforwardly coded by ourselves):

– *DeltaCon*: http://web.eecs.umich.edu/∼dkoutra/

– *MI-GRAAL*: http://www0.cs.ucl.ac.uk/staff/natasa/MI-GRAAL/index.html. The code was customized to consider as nodes similarities the degree, the clustering coefficient, and the betweenness centrality.

– *Graphlet-based measures*: the code for GCD-11 is at http://www0.cs.ucl.ac.uk/staff/natasa/GCD/index.html, the code for DCGD-129 is at http://www0.cs.ucl.ac.uk/staff/natasa/DGCD/index.html.

– *Portrait Divergence*: https://github.com/bagrow/portrait-divergence

– *NetLSD*: https://github.com/xgfs/NetLSD. The code was used in default mode (heat kernel, no normalization).

S3 Network models

We used three different network models for testing, namely Erdős-Rényi[3] (ER), Barabási-Albert[4] (BA) and Lancichinetti-Fortunato-Radicchi[5,6] (LFR). Tables S1 and S2 summarize the values of the parameters used to generate instances of each network model. The ER graphs were generated by setting the proper number of edges to achieve the desired densities. For the BA graphs, we tuned the number of edges to add at each step of the algorithm to get as close as possible to the required densities.

The LFR networks were generated with the algorithm and code by Lancichinetti, Fortunato and Radicchi[5,6]; both the degree and the community size distributions are assumed to be power law with exponent γ and β, respectively, and a parameter μ is set to tune the strength of the community structure: each node will share a fraction μ of its links with nodes outside its community, thus lower values of μ denote graphs with stronger community structure. The generation of an instance of an undirected LFR graph is realised by an algorithm whose main steps are the following:

– The degree of each node is assigned, from a power law distribution with exponent γ.

– The size of each community is assigned, from a power law distribution with exponent β.

– Nodes are randomly assigned to communities with an iterative procedure.

– Some rewiring steps are performed to enforce the condition on the fraction of links μ shared by each node inside its community.

The available code does not produce a network with the exact required number of edges, so we run it multiple times and took the network with the number of edges closest to the prescribed value. What ”close” means is specified by the *Edge tolerance* parameter reported in Tables S1 and S2, so that the number of edges of the generated network belongs to the interval [*Required edges* ± *Edge tolerance*].

As for directed networks, for an ER graph the direction of edges is chosen at random with equal probability. In the case of BA graphs, at each algorithm’s step a new vertex with the chosen fixed number of out-links is added, each link pointing to an already existing node with probability proportional to the in-degree of that node; this yields a BA network with hubs having large in-degree. Finally, the generation of a LFR network follows the same algorithm presented above, with the only difference that in the first step nodes are assigned the in-degree from a power law distribution with exponent γ and the out-degree from a δ distribution. The constraints needed in the undirected case are generalized to fit the directed case[6].

The numerical values of the parameters used to generate both undirected and directed networks are reported in Tables S1 and S2 for networks with 1 000 and 2 000 nodes, respectively.

Table S1. Parameters of the networks with 1 000 nodes used in the perturbation tests

	Undirected		Directed	
**Density**	**0.01**	**0.05**	**0.01**	**0.05**
Nodes	1 000	1 000	1 000	1 000
Edges	4 995	24 975	9 990	49 950

(a) Erdős-Rényi graphs

	Undirected		Directed	
**Density**	**≈0.01**	**≈0.05**	**≈0.01**	**≈0.05**
Nodes	1 000	1 000	1 000	1 000
Edges added in each step	5	25	10	51
Resulting edges	4 985	24 675	9 945	49 674

(b) Barabási-Albert graphs

	Undirected		Directed	
**Density**	**≈0.01**	**≈0.05**	**≈0.01**	**≈0.05**
Nodes	1 000	1 000	1 000	1 000
Exponent of degree distrib. (γ)	3	3	3	3
Exponent of community size distrib. (β)	1	1	1	1
Mean degree	10	50	10	50
Maximum degree	100	250	450	800
Mixing parameter (μ)	0.2	0.2	0.2	0.2
Minimum degree in community	5	5	5	5
Required edges	4 995	24 975	9 990	49 950
Edge tolerance	20	25	50	100

(c) LFR graphs: for directed graphs, mean and maximum degree refer to in-degree.

Table S2. Parameters of the networks with 2 000 nodes used in the perturbation tests

	Undirected		Directed	
**Density**	**0.01**	**0.05**	**0.01**	**0.05**
Nodes	2 000	2 000	2 000	2 000
Edges	19 990	99 950	39 980	199 900

(a) Erdős-Rényi graphs

	Undirected		Directed	
**Density**	**≈0.01**	**≈0.05**	**≈0.01**	**≈0.05**
Nodes	2 000	2 000	2 000	2 000
Edges added in each step	10	51	20	103
Resulting edges	19 945	100 674	39 790	200 644

(b) Barabási-Albert graphs

	Undirected		Directed	
**Density**	**≈0.01**	**≈0.05**	**≈0.01**	**≈0.05**
Nodes	2 000	2 000	2 000	2 000
Exponent of degree distrib. (γ)	3	3	3	3
Exponent of community size distrib. (β)	1	1	1	1
Mean degree	20	100	20	100
Maximum degree	900	1450	1100	1600
Mixing parameter (μ)	0.2	0.2	0.2	0.2
Minimum degree in community	5	5	5	5
Required edges	19 990	99 950	39 980	199 900
Edge tolerance	50	100	100	200

(c) LFR graphs: for directed graphs, mean and maximum degree are intended as mean and maximum in-degree.

S4 Perturbation tests: results for networks with density 0.05

Undirected networks

Figures S1 and S2 show the results of the perturbation tests on undirected networks with 0.05 edge density. The results are essentially equivalent to those obtained for networks with 0.01 density and described in the main paper: among other features, we again highlight the different behaviour of KNC and UNC methods, the fact that the LFR curve almost always stays above the other curves in the KNC methods, and the high variability of GCD-11. The diameter proves completely inadequate as a distance. The only qualitative difference with respect to the 0.01 case concerns the PDIV distance: the initial step is not as sharp as before, and almost all curves, with the exception of the ER curve in the *pADD-test* and *pREM-test* and of the LFR curve in the *dSWI-test*, saturate immediately. In conclusion, in the undirected case a change in the density of the graphs does not seem to heavily influence the behaviour of the results of the different methods.

Directed networks

Figures S3 to S5 show the results of the perturbation tests on directed networks with 0.05 edge density. We essentially observe the same qualitative results as in the 0.01 density case. The DGCD-129 distance presents now a pronounced step in the first few perturbations, except for the *pREM-test*. As for the undirected case, it does not seem that the methods for directed networks are influenced by changes in the network density.

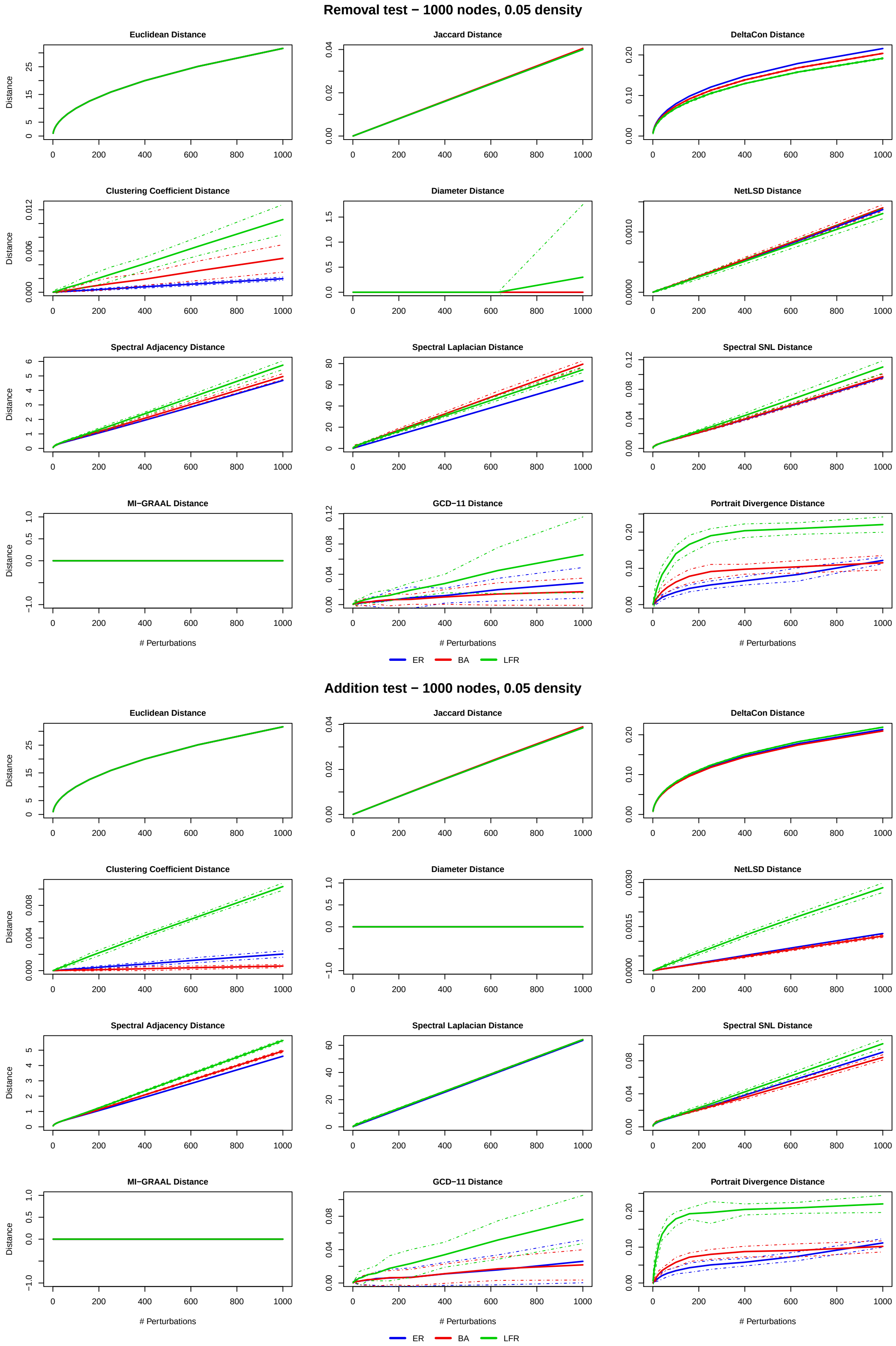

Figure S1. Perturbations tests: results of the *Removal (pREM-test)* and *Addition (pADD-test)* tests for the 12 distances and the 3 undirected models ER, BA, LFR (density 0.05). Solid (dashed) lines are the mean (±3 std) values obtained over the 10 replications of the perturbation histories.

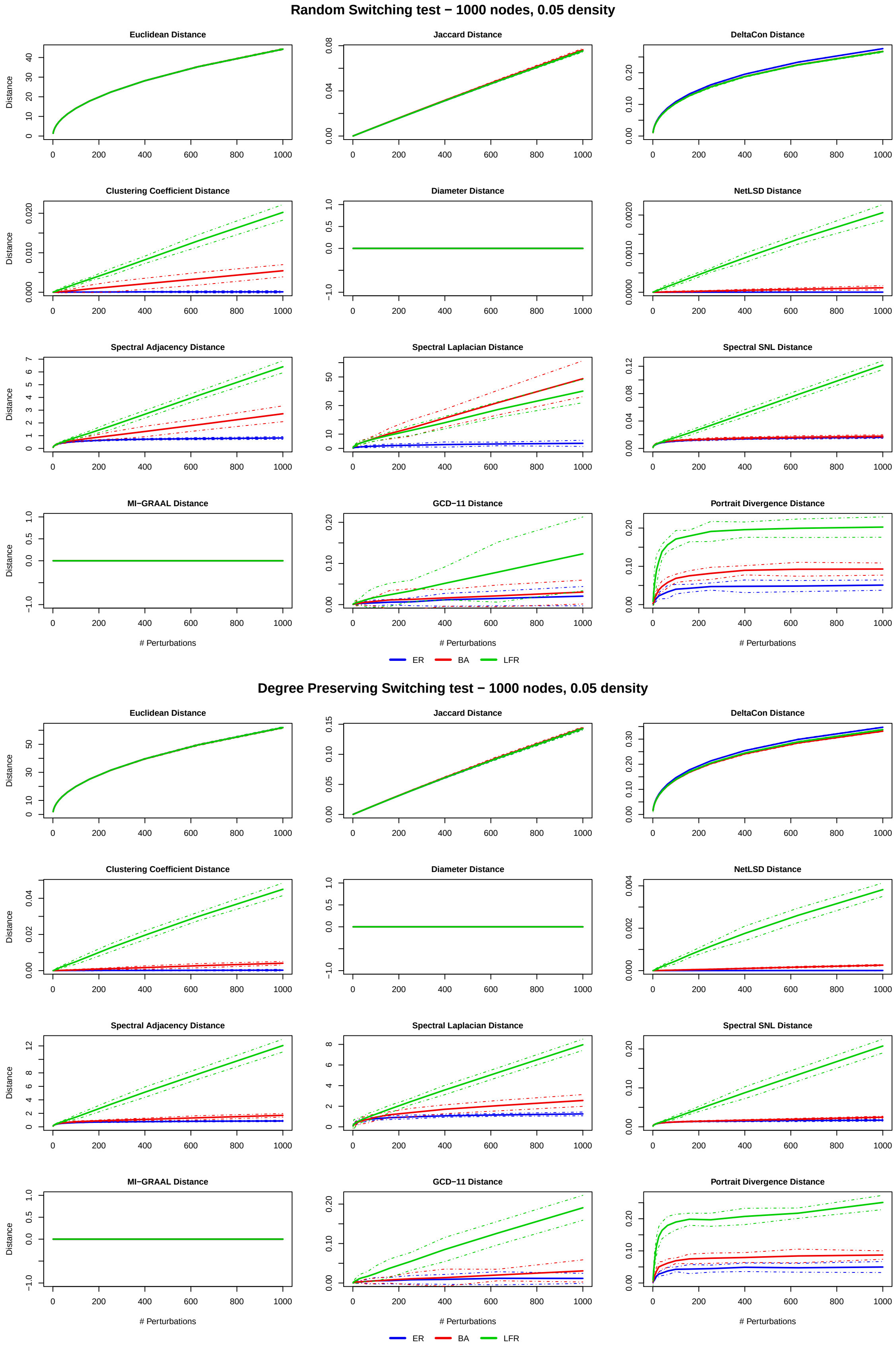

Figure S2. Perturbations tests: results of the *Random switching (pRSW-test)* and *Degree-preserving switching (pDSW-test)* test for the 12 distances and the 3 undirected models ER, BA, LFR (density 0.05). Solid (dashed) lines are the mean (±3 std) values obtained over the 10 replications of the perturbation histories.

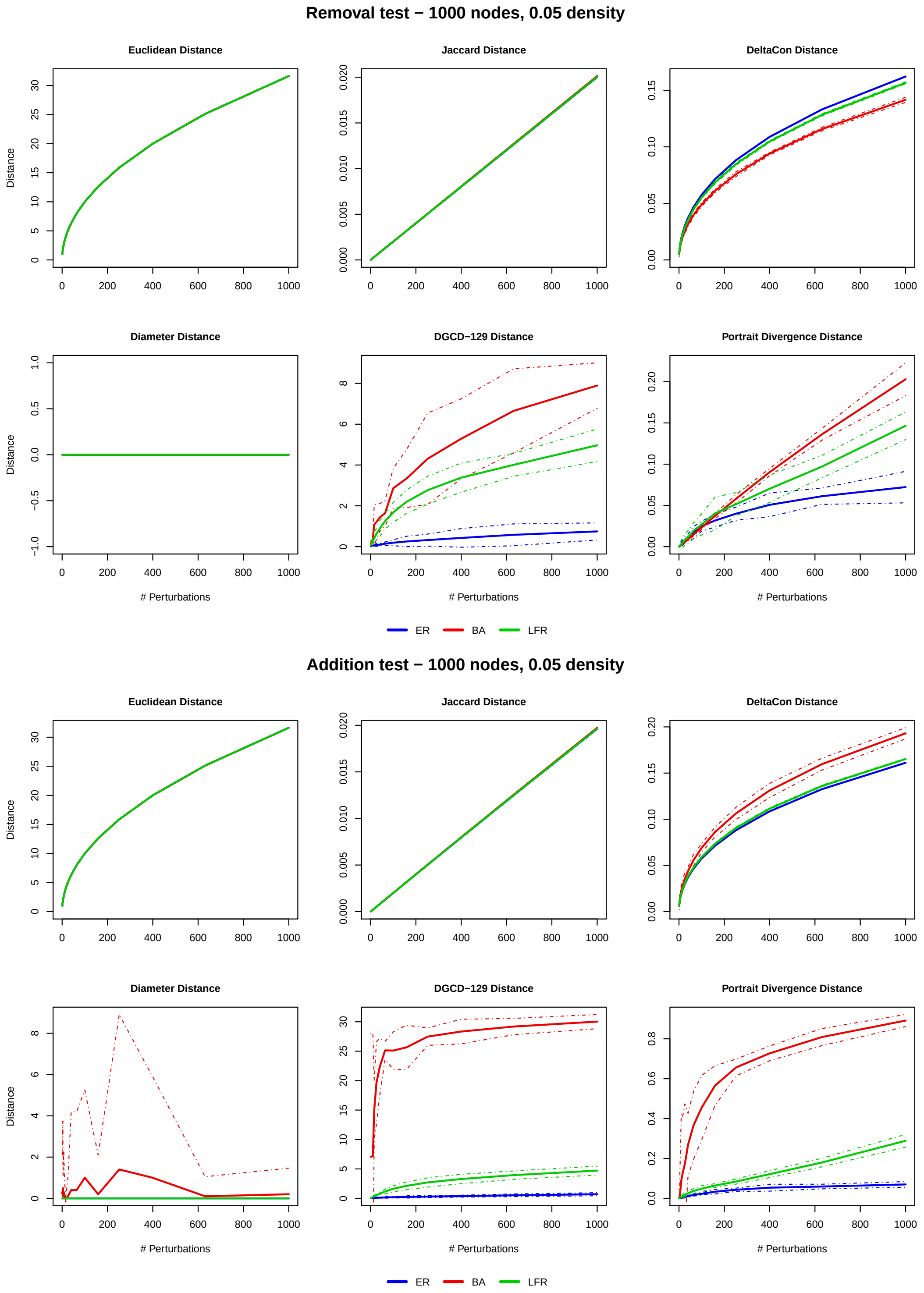

Figure S3. Perturbations tests: results of the *Removal (pREM-test)* and *Addition (pADD-test)* tests for the 6 distances and the 3 directed models ER, BA, LFR (density 0.05). Solid (dashed) lines are the mean ($\pm$3 std) values obtained over the 10 replications of the perturbation histories.

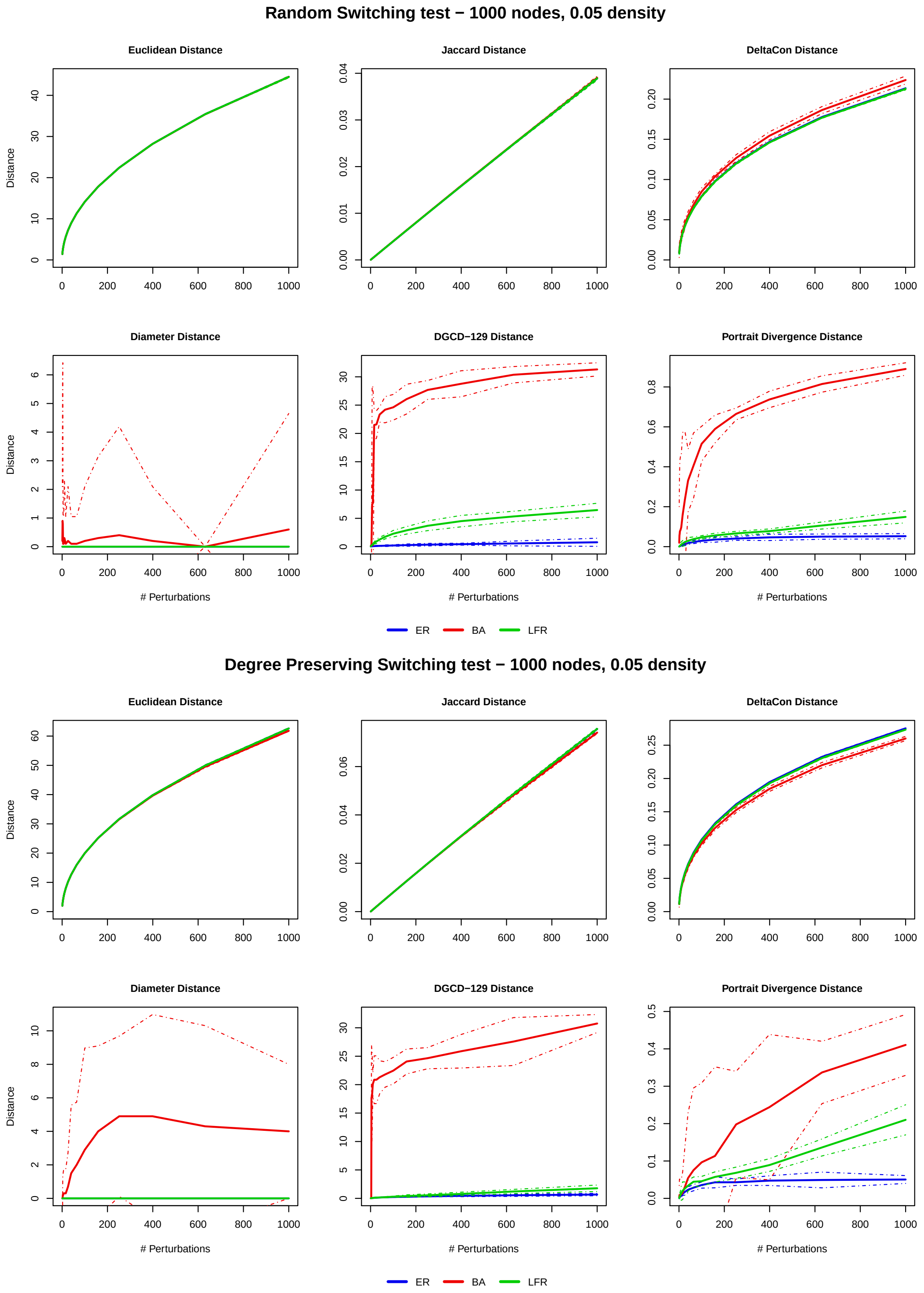

Figure S4. Perturbations tests: results of the *Random switching (pRSW-test)* and *Degree-preserving switching (pDSW-test)* test for the 6 distances and the 3 directed models ER, BA, LFR (density 0.05). Solid (dashed) lines are the mean (± 3 std) values obtained over the 10 replications of the perturbation histories.

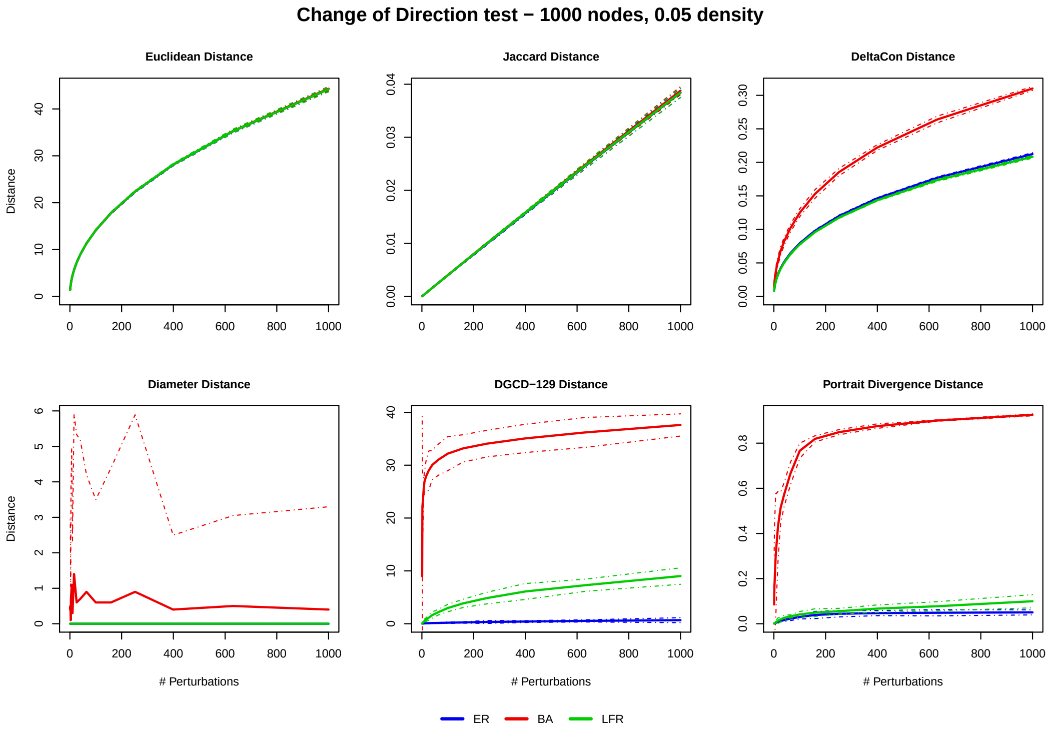

Figure S5. Perturbations tests: results of the *Change of direction (pDIR-test)* test for the 6 distances and the 3 directed models ER, BA, LFR (density 0.01). Solid (dashed) lines are the mean (±3 std) values obtained over the 10 replications of the perturbation histories.

S5 Clustering tests: dendrograms for undirected networks

In Figure S6 we show the dendrograms for the clustering tests on undirected networks that were not included in the main paper. As already pointed out, these three methods are able to group network of the same class, but only if they have the same size and density.

In Tables S3a and S4a we report the values of the AUPR metric obtained from the Precision-Recall analysis for undirected and directed networks, respectively. To verify that the methods perform well in grouping networks of similar size and density, we repeated the test for each one of the four size/density subsets. The results are in Tables S3b and S4b.

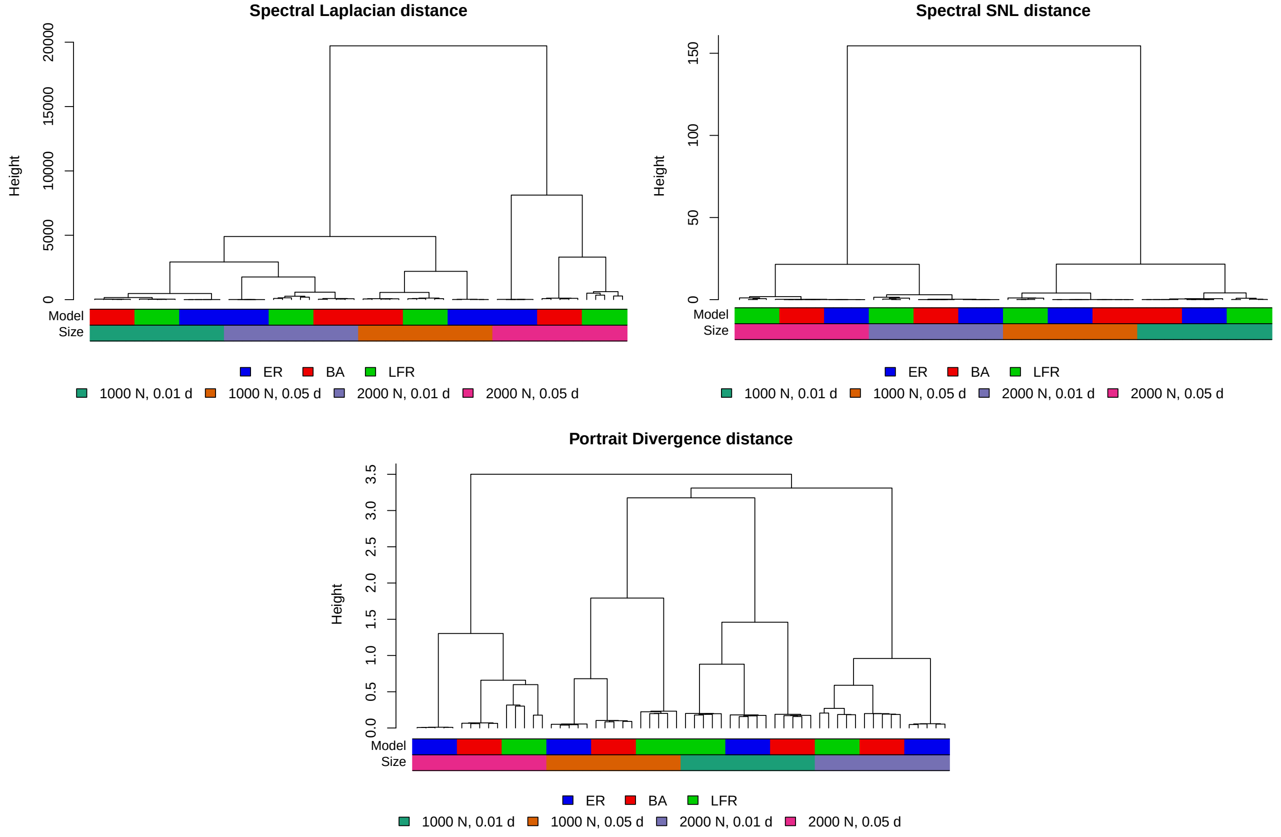

Figure S6. Clustering tests: the dendrograms, for undirected networks, of the methods not included in Fig. 5 of the main text.

Table S3. AUPR values obtained from the Precision-Recall analysis for the clustering tests on undirected networks

(a) AUPR values when all 60 networks are considered

Distance	AUPR
Spectral Adjacency	0.458
Spectral Laplacian	0.471
Spectral SNL	0.391
Clustering coefficient	0.620
GCD-11	0.688
NetLSD	0.386
Portrait Divergence	0.455
Random classifier	0.322

(b) AUPR values when only networks with same size and density are considered. The AUPR of a random classifier is 0.286 in all cases.

Distance	AUPR
Spectral Adjacency	1
Spectral Laplacian	1
Spectral SNL	0.806
Clustering coefficient	1
GCD-11	1
NetLSD	0.904
Portrait Divergence	1

1000 nodes, 0.01 density

Distance	AUPR
Spectral Adjacency	0.985
Spectral Laplacian	1
Spectral SNL	0.833
Clustering coefficient	1
GCD-11	0.854
NetLSD	0.832
Portrait Divergence	1

1000 nodes, 0.05 density

Distance	AUPR
Spectral Adjacency	0.996
Spectral Laplacian	0.969
Spectral SNL	0.834
Clustering coefficient	0.915
GCD-11	1
NetLSD	0.675
Portrait Divergence	0.995

2000 nodes, 0.01 density

Distance	AUPR
Spectral Adjacency	0.854
Spectral Laplacian	1
Spectral SNL	0.782
Clustering coefficient	0.856
GCD-11	0.816
NetLSD	0.573
Portrait Divergence	0.860

2000 nodes, 0.05 density

Table S4. AUPR values obtained from the Precision-Recall analysis for the clustering tests on directed networks

(a) AUPR values when all 60 networks are considered

Distance	AUPR
DGCD-129	0.928
Portrait Divergence	0.685
Random classifier	0.322

(b) AUPR values when only networks with same size and density are considered. The AUPR of a random classifier is 0.286 in all cases.

Distance	AUPR
DGCD-129	1
Portrait Divergence	1

1000 nodes, 0.01 density

Distance	AUPR
DGCD-129	1
Portrait Divergence	1

1000 nodes, 0.05 density

Distance	AUPR
DGCD-129	1
Portrait Divergence	0.995

2000 nodes, 0.01 density

Distance	AUPR
DGCD-129	1
Portrait Divergence	1

2000 nodes, 0.05 density

Tests on real-world networks

European Air Transportation Network. The European Air Transportation dataset was described and analysed by Cardillo *et al.*[7]. We used a preprocessed version of the dataset downloaded from https://comunelab.fbk.eu/data.php, updated at 2011 and composed of 450 nodes representing airports. Each of them is labelled with the ICAO airport code, and latitudes and longitudes are reported. We found two nodes labelled as ”XXXX” and ”YYYY”, both with zero latitude and longitude: we removed them from the dataset. Table S5 lists all the airlines considered in the dataset.

Table S5. List of the 37 airlines in the European Air Transportation dataset

1	Lufthansa	20	LOT Polish Airlines
2	Ryanair	21	Vueling
3	Easyjet	22	Air Nostrum
4	British Airways	23	Air Lingus
5	Turkish Airlines	24	Germanwings
6	Air Berlin	25	Panagra Airways
7	Air France	26	Netjets
8	Scandinavian Airlines	27	Transavia Holland
9	KLM	28	Niki
10	Alitalia	29	SunExpress
11	Swiss International Airlines	30	Aegean Airlines
12	Iberia	31	Czech Airlines
13	Norwegian Air Shuttle	32	European Air Transport
14	Austrian Airlines	33	Malev Hungarian Airlines
15	Flybe	34	Air Baltic
16	Wizz Air	35	Wideroe
17	TAP Portugal	36	TNT Airways
18	Brussels Airlines	37	Olympic Air
19	Finnair		

The dendrograms resulting from the cluster analysis are shown in Figure S7. For the discussion of the results, see the main paper.

FAO Trade Network. The FAO dataset was downloaded from https://comunelab.fbk.eu/data.php and integrated with information about products using the official FAO website http://www.fao.org/faostat/en/#data/TM. The dataset was described and analysed by De Domenico et al.[8]. It represents the worldwide food import/export multilayer network updated at 2010. The network is directed and weighted, the weights representing the export value, in thousands of USD, of a product from a country to another one.

We denote by $E = [e_{cp}]$ the country/product trade matrix, whose entry e_{cp} is the export value of product p by country c. Then the Revealed Comparative Advantage RCA_{cp} for product p in country c is given by

$$RCA_{cp} = \frac{\frac{e_{cp}}{\sum_{p'} e_{cp'}}}{\frac{\sum_{c'} e_{c'p}}{\sum_{c'p'} e_{c'p'}}}. \tag{S1}$$

The results in the main paper refer to the binarized version of the network, which is obtained, for each product p, by retaining only the links departing from countries c having $RCA_{cp} > 1$, i.e., countries which are significant exporters of that product[9, 10].

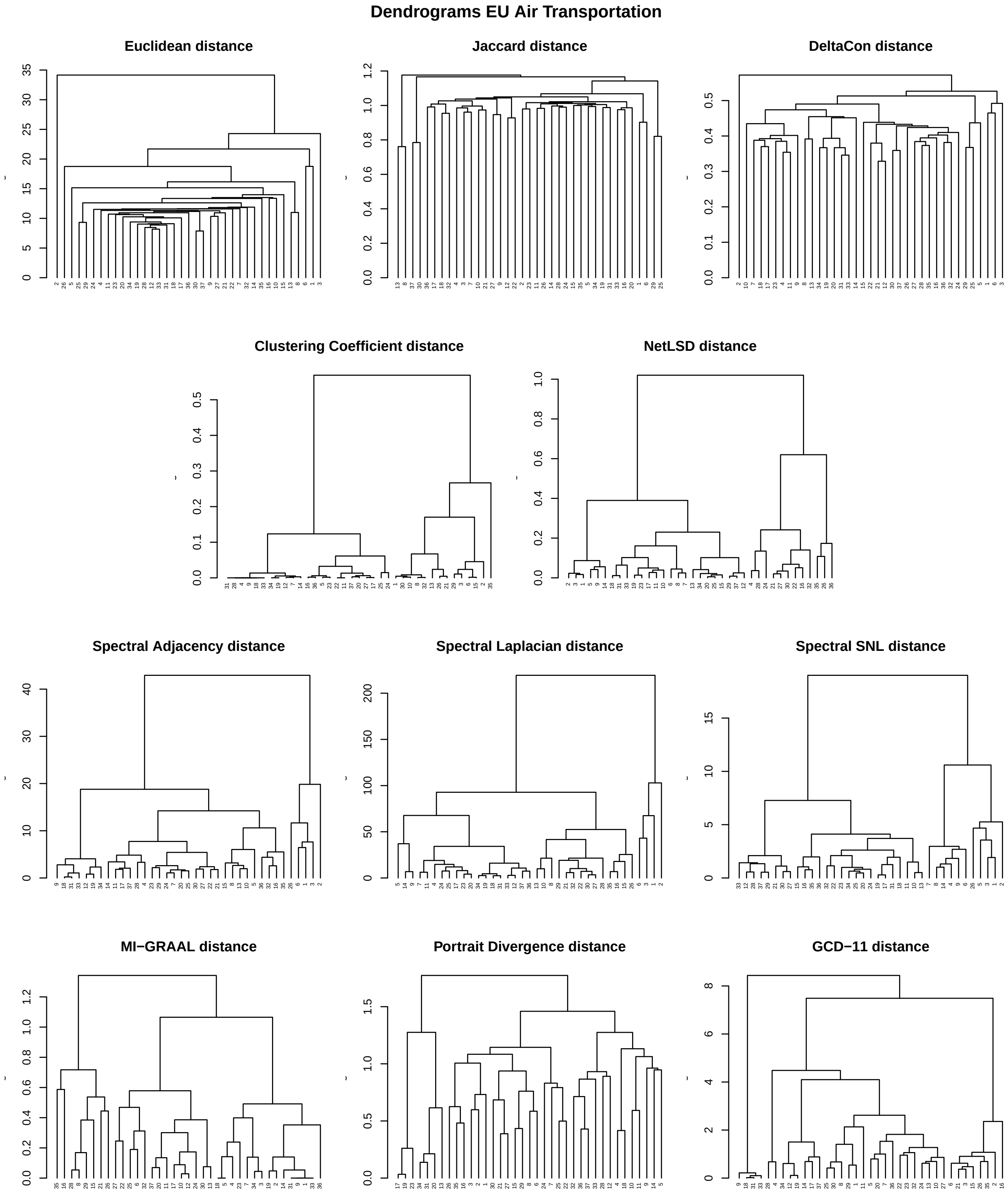

Figure S7. Dendrograms for the cluster analysis of the European Air Transportation Network

References

1. Ioffe, S. Improved consistent sampling, weighted minhash and l1 sketching. In *Proc. 2010 IEEE Int. Conf. Data Mining*, 246–255 (2010).
2. Chierichetti, F., Kumar, R., Pandey, S. & Vassilvitskii, S. Finding the Jaccard median. In *Proc. 21st Annual ACM-SIAM Symposium on Discrete Algorithms*, 293–311 (SIAM, 2010).
3. Erdös, P. & Rényi, A. On random graphs, I. *Publ. Math.-Debr.* **6**, 290–297 (1959).
4. Barabasi, A.-L. & Albert, R. Emergence of scaling in random networks. *Science* **286**, 509–512 (1999).
5. Lancichinetti, A., Fortunato, S. & Radicchi, F. Benchmark graphs for testing community detection algorithms. *Phys. Rev. E* **78**, 046110 (2008).
6. Lancichinetti, A. & Fortunato, S. Benchmarks for testing community detection algorithms on directed and weighted graphs with overlapping communities. *Phys. Rev. E* **80**, 016118 (2009).
7. Cardillo, A. *et al.* Emergence of network features from multiplexity. *Sci. Rep.* **3**, 1344 (2013).
8. Domenico, M. D., Nicosia, V., Arenas, A. & Latora, V. Structural reducibility of multilayer networks. *Nat. Commun.* **6**, 6864 (2015).
9. Balassa, B. Trade liberalisation and revealed comparative advantage. *The Manchester School* **33**, 99–123 (1965).
10. Piccardi, C. & Tajoli, L. Complexity, centralization and fragility in economic networks. *PLoS ONE* **13**, e0208265 (2018).
